# Design, Synthesis, Antimicrobial Evaluation, Molecular Docking, and Computational Studies of New 1,2,4-Triazolo[4,3-a]pyrimidin-5(1 H)-one Derivatives

**DOI:** 10.1007/s10895-025-04412-w

**Published:** 2025-06-30

**Authors:** Abd-Allah S. El-Etrawy, Noha Ryad, Amr Fouda, Farag F. Sherbiny, Adel. A. H. Abdel-Rahman, Mahmoud A. S. Sakr

**Affiliations:** 1 Department of Chemistry, Center of Basic Science (CBS), Misr University for Science and Technology (MUST), Al-Motamayez District, 6th of the October City 77, Giza, Egypt; 2 Pharmaceutical Organic Chemistry Department, College of Pharmaceutical Sciences and Drug Manufacturing, Misr University for Science and Technology (MUST), 6th of October City, P.O. Box 77, Giza, Egypt; 3 Botany and Microbiology Department, Faculty of Science, Al-Azhar University, Nasr City, Cairo 11884 Egypt; 4 Pharmaceutical Organic Chemistry Department, Faculty of Pharmacy (Boys), Al-Azhar University, Cairo, 11884 Egypt; 5 Department of Chemistry, Faculty of Science, Menoufia University, Shebin El-Koam, Egypt

**Keywords:** [1,2,4] triazolo[4,3-a] Pyrimidine, Antimicrobial activity, DNA gyrase B, Molecular docking, DFT, TD-DFT

## Abstract

This study reports the synthesis and comprehensive characterization of a new series of 1,2,4-triazolo[4,3-a] pyrimidin-5-(1*H*)-one derivatives. These compounds were synthesized from 2-hydrazinopyrimidines via cyclization reactions. Density Functional Theory (DFT) calculations revealed the molecular structures, stability, and electronic properties of the derivatives. Computational studies revealed that the compounds exhibited planar conformations, though the triazole rings were found to be non-coplanar with the pyrimidine ring. Substituents like methyl, amino, and propyl groups influenced bond lengths, dihedral angles, and electronic properties. Binding energies confirmed molecular stability, while IR spectra supported dynamical stability. UV-Vis’s absorption spectra matched experimental data, showing maxima in the 250–400 nm range, with redshifts attributed to electron-donating substituents. Density of State (DOS) and HOMO/LUMO analyses indicated strong orbital interactions and electron delocalization. Molecular docking studies suggested favorable binding with the DNA gyrase receptor, indicating potential antimicrobial activity against both Gram-positive and Gram-negative bacteria. This study provides valuable insights into the synthesis, structural, electronic, and antimicrobial properties of 1,2,4-triazolo[4,3-a] pyrimidin-5-(1*H*)-one derivatives, highlighting their potential applications in medicinal chemistry and materials science.

**Supplementary Information:**

The online version contains supplementary material available at 10.1007/s10895-025-04412-w.

## Introduction

Fused pyrimidine derivatives, along with their diverse heterocyclic scaffolds, have a rich history and play significant roles in various fields, ranging from their discovery as crucial heterocyclic ring systems in nucleic acids to their current applications in chemotherapy and antimicrobial activities. These compounds exhibit a wide range of pharmacological properties, including anti-inflammatory [1, 2], antimicrobial [3–5], anti-HIV [6, 7], anti-malarial [8–10], anticancer [11, 12], antimycobacterial [13–16], and antiangiogenic [17, 18] activities. Among the fused pyrimidines, 1,2,4-triazolo[4,3-*a*] pyrimidinones have garnered attention due to their reported calcium channel-blocking effects and potential applications in treating conditions such as hypertension, cardiovascular diseases [19, 20]. In addition, some studies have explored their potential effects on anxiety symptom, although this remains an off-label and less-established area of research [21]. Despite significant efforts to understand and combat microbial infections, antimicrobial resistance remains a major challenge in modern medicine. “It is known that the major difference between Gram-positive and Gram-negative bacteria lies in their cell wall structure: Gram-positive bacteria have a thick peptidoglycan layer, while Gram-negative bacteria have a thin peptidoglycan layer and an outer membrane. Additionally, Gram-positive bacteria often produce exotoxins, whereas Gram-negative bacteria can produce both endotoxins (lipopolysaccharides) and exotoxins.” Antibiotics are increasingly losing their effectiveness as drug resistance spreads globally, leading to more challenging-to-treat infections. Inadequate treatment of infectious diseases, over-prescription of antibiotics, and their improper use by patients have contributed to the development of drug-insensitive microorganisms. This situation not only complicates the treatment of infectious diseases but also exacerbates antibiotic resistance [22]. The rise of antibiotic resistance can be attributed to the overuse and misuse of drugs, which poses a significant risk and cost in the treatment of infectious diseases, particularly during medical procedures such as surgery [23]. To overcome antibiotic resistance, the development of novel medicinal compounds that are effective against infections has become a crucial area of scientific research. The 1,2,4-triazole motif, with its unique characteristics, offers promising opportunities for researchers to advance novel drug candidates with improved efficacy and selectivity [24].

In recent years, triazolo[4,3-a] pyrimidin-5-(1*H*)-one derivatives have garnered significant attention due to their diverse biological activities and potential applications in medicinal chemistry. Previous studies have demonstrated the synthesis and characterization of various heterocyclic compounds with promising pharmacological properties (e.g., antimicrobial, anticancer) [25]. Further research has explored the structural modifications and their impact on the biological efficacy of these compounds [26, 27]. Additionally, the adsorption properties of related compounds have been investigated, revealing insights into their potential for chemical sensing and catalysis [28, 29]. In this study, our focus was on synthesizing a novel series of 1,2,4-triazole derivatives incorporating various functional groups known for their antibacterial and antifungal activities. Subsequently, we evaluated these newly synthesized compounds to determine their antimicrobial potential against a wide range of gram-negative and gram-positive bacteria, as well as fungi. Additionally, we conducted molecular docking studies to gain insights into the interactions between the synthesized compounds and their respective target receptors or enzymes. Furthermore, we performed important quantum studies to investigate the electronic and optical properties of the novel series of 1,2,4-triazole derivatives. These calculations were carried out using density functional theory (DFT) and time-dependent DFT (TD-DFT) methods, specifically employing the CAM-B3LYP/6-311G++ (d, p) level of theory. By conducting these quantum calculations, we aimed to elucidate the electronic structures, energy levels, and optical properties, such as UV-Vis’s absorption spectra, of the synthesized compounds. This information provides valuable insights into the potential applications and mechanisms of action of the derivatives, aiding in the design and development of novel compounds with improved efficacy and selectivity. Overall, this comprehensive study integrates organic synthesis, antimicrobial evaluation, molecular docking, and quantum calculations to explore the antimicrobial potential and understand the electronic and optical properties of the newly synthesized 1,2,4-triazole derivatives.

### Rationale Drug Design

**Fig. 1:**
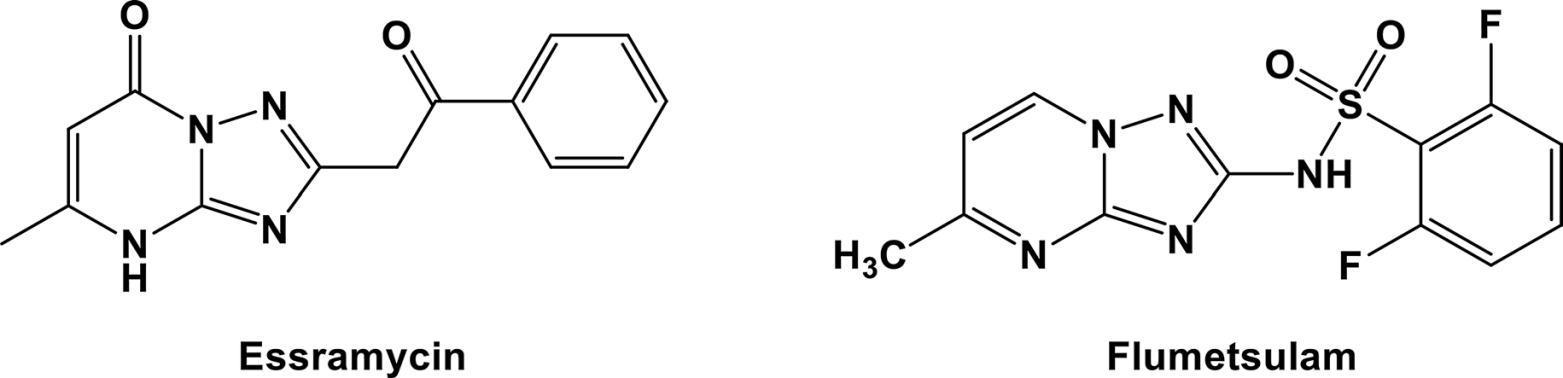
The basic structural feature requirements for triazolo-pyrimidine moiety as reported

The structural resemblance of triazolo-pyrimidine to the purine ring, as well as its similarity to other aza-indolizines, positions it as a potential bio-isostere for purine derivatives. By replacing the purine ring with triazolo-pyrimidine, it is possible to retain desirable physicochemical properties within favorable ranges, including water solubility and permeability, while significantly supporting biological activities [30]. Essramycin, a natural product isolated from the Egyptian Mediterranean coast, is an example of a triazolo-pyrimidine compound with relatively potent and broad-spectrum antibacterial activities against both Gram-positive and Gram-negative bacteria [31]. Moreover, compounds such as flumetsulam, initially developed and marketed as herbicides, have demonstrated anti-TB activities, showcasing the potential therapeutic applications of triazolo-pyrimidines (see Fig. 1) [32]. Consequently, there is a continued and intensified focus on discovering new therapeutic targets for treating infectious diseases. Triazolo-pyrimidine has emerged as a promising candidate for the development of novel antibacterial and antifungal agents [33]. Its exploration as a drug target holds significant potential for combating drug-resistant pathogens and addressing the ongoing challenges associated with infectious diseases.

**Fig. 2:**
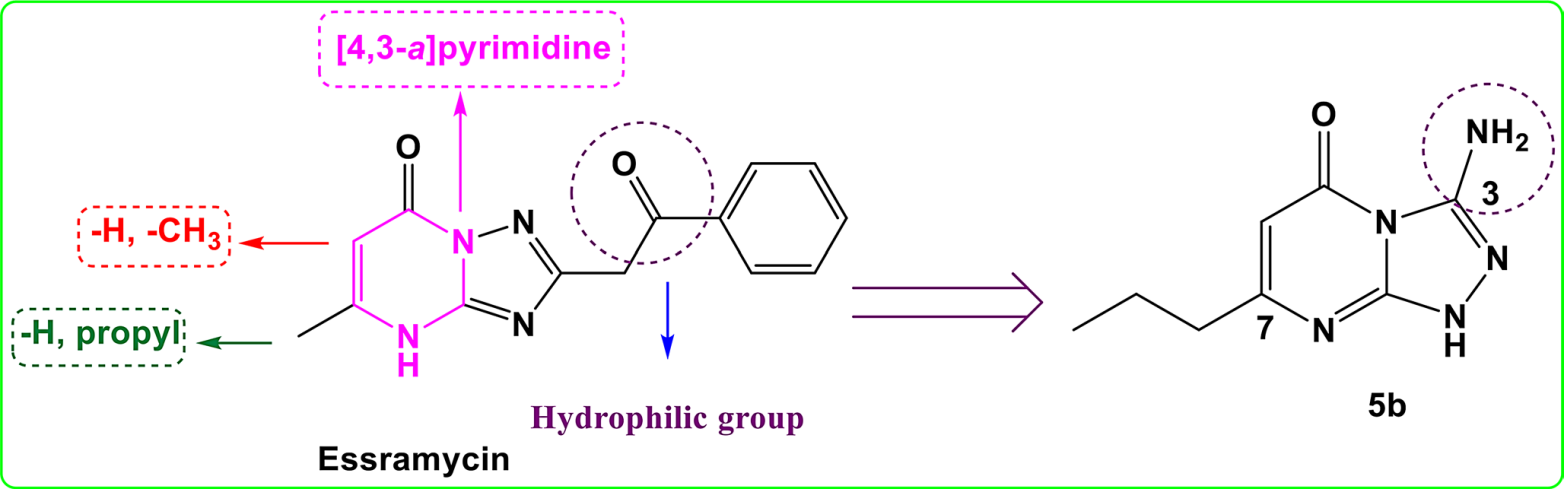
Planned design of novel triazolopyrimidine derivatives for antimicrobial activity

To identify potent antimicrobial activity as a candidate treatment for infectious disease, our approach was to design new compounds through structural optimization with improved potency against the targets using the reference drug essramycin. Thus, we expect that the hybridization of structural features of triazolopyrimidine and hydrophobic moieties will result in newly synthesized hybrids that may maintain the overall structural features required to bind with critical amino acid residues. The triazolopyrimidine system is a privileged scaffold in medicinal chemistry. Thus, the essential design was prepared by chemical pharmacophoric modifications, including bioisosteric chemical features of triazolopyrimidine and introducing an alkyl group at the 6-, 7-, 3,6-, or 3,7-positions. Additionally, introducing the amino group and alkyl group at the 3,6- or 3,7-position in the triazolopyrimidine nucleus to improve and explore the chemical features, including hydrophobic and hydrophilic moieties around the triazolopyrimidine scaffold (See Fig. 2).

## Experimental

### General

All the chemicals used were commercially available and they were used without any further purification. The solvents utilized were purified and freshly distilled prior to use, following standard procedures. To monitor the progress of the reaction mixtures, thin-layer chromatography (TLC) was employed. The TLC plates were visualized using a UV lamp at a wavelength of 254 nm. Melting points of the synthesized compounds were determined using a Thermo Fisher Scientific instrument. Infrared (IR) spectra were recorded using a Bruker tensor 27 FT-IR Spectrophotometer. Proton nuclear magnetic resonance (^1^H NMR) and carbon-13 nuclear magnetic resonance (^13^C NMR) spectra were obtained using Bruker spectrophotometers operating at frequencies of 400 & 500 MHz and 100 & 125 MHz, respectively. Chemical shifts (δ) are reported in parts per million (ppm), and tetramethyl silane (TMS) was used as the internal standard. The mass spectra were run on a Shimadzu Qp 5050 Ex Spectrometer. The microanalyses for C, H and N were performed on Perkin–Elmer elemental analyzer. The antimicrobial evaluation was carried at the Microbiology Department, Faculty of Science, Al-Azhar University, Nasr City, Cairo11884, Egypt.

### General Procedure: Synthesis of 2-hydrazenopyrimidin-4(3*H*)-one Derivatives 2a, B

A mixture of 2-thioxo-2,3-dihydropyrimidin-4(*1 H*)-ones 1a (0.1 mol 14.2 g) or 1b (0.1 mol 17.0 g) and hydrazine hydrate 99% (30 mL) in of absolute ethanol (100 mL) was heated under reflux for 12 h. The reaction mixture was then cooled and the solid products obtained were filtered off, dried, and recrystallized from ethanol to give pure 2a, b.

#### 2-Hydrazino-5-Methylpyrimidin-4(3*H*)-one (2a)

Yield (13.3 g, 95%), solid white crystal m.p. 223–225 ºC., R_*f*_ = 0.44 (10% MeOH/CH_2_Cl_2_); IR υ (cm^− 1^): 3319 and 3279 (NH_2_), 3211 (NH), 1670 (C = O), 1616 (C = N), 1544, 1487 (C = C ring stretch.); ^1^H NMR (400 MHz, DMSO-*d*_*6*_) δ (ppm): 1.78 (s, 3 H, CH_3_); 3.41 (brs, 1H, NH_2,_ D_2_O exchangeable); 7.12, 7.13 (brs, 2 H, NH. D_2_O exchangeable), 7.14 (s, 1H, H-6); ^13^C NMR (100 MHz, DMSO-*d*_*6*_), δ (ppm): 13.0, 98.1, 129.5, 159.9; MS (EI): *m*/*z* = 140.11 [M^+^], anal. calcd. for C_5_H_8_N_4_O (140.14.): C, 42.85; H, 5.75; N, 39.98. Found C, 42.95; H, 5.67; N, 39.88%.

#### 2-Hydrazinyl-6-propylpyrimidin-4(3*H*)-one (2b)

Yield (15.5 g, 92%), solid white crystal m.p. 168–170 ºC., R_*f*_ = 0.4 (10% MeOH/CH_2_Cl_2_); IR υ (cm^− 1^): 3348 and 3269 (NH_2_), 3213 (NH), 1639 (C = O), 1633 (C = N), 1571, 1421 (C = C ring stretch.); ^1^H NMR (400 MHz, DMSO-*d*_*6*_) δ (ppm): 0.78 (t, 3 H, CH_3_, *J* = 7.5 Hz), 1.49–1.54 (m, 2 H, CH_2_), 2.20 (t, 2 H, CH_2_, *J* = 7.5 Hz), 3.95 (brs, 1H, NH_2,_ D_2_O exchangeable), 5.34 (s, 1H, *H*_*5*_); 1.08, 7.15 (brs, 2 H, 2NH. D_2_O exchangeable); ^13^C NMR (100 MHz, DMSO-*d6*), δ (ppm): 14.0, 21.1, 45.1, 99.9, 157.9, 161.0, 163.7; MS (EI): *m*/*z* = 169.20 [M^+^ + 1], anal. calcd. for C_7_H_12_N_4_O (168.20): C, 49.99; H, 7.19; N, 33.31. Found C, 50.09; H, 7.15; N, 33.25%.

### General Procedure: Synthesis of 6- and 7-disustituted of 3-methyl- [1,2,4] triazolo[4,3-*a*] pyrimidin-5(1*H*)-one 3a, B

A mixture of 2a (10 mmol, 1.4 g) or 2b (10 mmol, 1.68 g) in glacial acetic acid (30 mL) was heated under reflux for 6 h. After cooling the reaction mixture was poured onto ice cold water. The precipitate formed was collected by filtration, washed with water, dried and crystallized from ethanol to give the corresponding 3a, b in good yields.

#### 3,6-Dimethyl- [,2,4] triazolo[4,3-*a*] pyrimidin-5(1*H*)-one (3a)

Yield (1.4 g, 86%), solid white crystal m.p. 260–262 ºC., R_*f*_ = 0.29 (5% MeOH/CH_2_Cl_2_); IR (KBr, ν cm^− 1^): 3256 (NH), 3017 (aromatic CH stretch.), 2909, 2852 (aliphatic CH, stretch.), 1676, (CONH), 1626 (C = N), 1584, 1475 (C = C ring stretch.); ^1^H NMR (500 MHz, DMSO-*d6*), δ (ppm): 1.76 (s, 3 H, CH_3_), 1.87 (s, 3 H, CH_3_), 7.47 (s, 1H, *H*_*7*_), 9.7 (br,1H, NH); ^13^C NMR (125 MHz, DMSO-*d6*), δ (ppm): 12.7, 20.9, 100.2, 112.5, 152.8, 155.7, 168.3; MS (EI): *m*/*z* = 164.27 [M^+^], anal. calcd. for C_7_H_8_N_4_O (164.16): C, 51.21; H, 4.91; N, 34.13. Found C 51.40, H 4.45, N 33.79%.

#### 3-Methyl-7-propyl- [1,2,4] triazolo[4,3-*a*] pyrimidin-5(1*H*)-one (3b)

Yield (1.6 g, 81%), solid white crystal m.p. 240–242 ºC., R_*f*_ = 0.51 (5% MeOH/CH_2_Cl_2_); IR (KBr, ν cm^− 1^): 3305 (NH), 3035 (aromatic CH stretch.), 2968, 2941, 2876 (aliphatic CH, stretch.), 1687, (CONH), 1612 (C = N), 1570, 1484 (C = C ring stretch.); ^1^H NMR (500 MHz, DMSO-*d6*), δ (ppm): 0.89 (t, 3 H, CH_3_, *J* = 7.5 Hz), 1.54–1.61 (m, 2 H, CH_2_), 2.26 (t, 2 H, CH_2_, *J* = 7.5 Hz), 5.49 (s, 1H, *H*_*6*_), 9.65 (br,1H, NH); ^13^C NMR (125 MHz, DMSO-*d6*), δ (ppm): 14.0, 21.1, 21.4, 39.8, 101.4, 124.5, 156.7, 163.8, 169.4; MS (EI): *m*/*z* = 192.69 [M^+^], anal. calcd. for C_9_H_12_N_4_O (192.22): C, 56.24; H, 6.29; N, 29.15. Found C, 56.45; H, 6.19; N, 29.11%.

### General Procedure: Synthesis 6- and 7-disustituted of [1,2,4] triazolo[4,3-*a*] pyrimidin-5(1*H*)-one 4a, B

A mixture of 2a (10 mmol, 1.4 g) or 2b (10 mmol, 1.68 g) in triethyl orthoformate (40 mL) was heated under reflux for 4 h. After cooling the precipitate formed was collected by filtration, dried and crystallized from ethanol to give the corresponding 4a, b in good yields.

#### 6-Methyl- [1,2,4] triazolo[4,3-*a*] pyrimidin-5(1*H*)-one (4a)

Yield (1.3 g, 84%), solid white crystal m.p. 210–212 ºC., R_*f*_ = 0.36 (5% MeOH/CH_2_Cl_2_); IR (KBr, ν cm^− 1^): 3255 (NH), 3097 (aromatic CH stretch.), 2940, 2859 (aliphatic CH, stretch.), 1691, (CONH), 1608 (C = N), 1526, 1470 (C = C ring stretch.); ^1^H NMR (500 MHz, DMSO-*d6*), δ (ppm): 1.97 (s, 3 H, CH_3_), 7.93 (s, 1H, *H*_*7*_), 7.94 (br,1H, NH), 9.015 (s, 1H, *H*_*3*_); ^13^C NMR (125 MHz, DMSO-*d6*), δ (ppm): 12.4, 105.3, 133.0, 149.4, 156.9, 162.0; MS (EI): *m*/*z* = 151.19 [M^+^ + 1], anal. calcd. C_6_H_6_N_4_O (150.14): C, 48.00; H, 4.03; N, 37.32. Found C, 48.08; H, 4.00; N, 37.12%.

#### 7-Propyl- [1,2,4] triazolo[4,3-a] pyrimidin-5(*1 H*)-one (4b)

Yield (1.8 g, 89%), solid white crystal m.p. 146–148 ºC., R_*f*_ = 0.33 (5% MeOH/CH_2_Cl_2_); IR (KBr, ν cm^− 1^): 3397 (NH), 3126, 3032 (aromatic CH stretch.), 2957, 2930, 2873 (aliphatic CH, stretch.), 1699, (CONH), 1640 (C = N), 1577, 1466 (C = C ring stretch.); ^1^H NMR (500 MHz, DMSO-*d6*), δ (ppm): 0.90 (t, 3 H, CH_3_, *J* = 8.5 Hz), 1.61–1.70 (m, 2 H, CH_2_), 2.52 (t, 2 H, CH_2,_*J* = 9 Hz), 5.67 (s, 1H, *H*_*6*_), 8.51 (br,1H, NH) 8.98 (s, 1H, *H*_*3*_); MS (EI): *m*/*z* = 178.18 [M^+^], anal. calcd. for C_8_H_10_N_4_O (178.19): C, 53.92; H, 5.66; N, 31.44. Found C, 53.95; H, 5.61; N, 31.41%.

### General Procedure: Synthesis 6- and 7-disustituted of 3-amino- [1,2,4] triazolo[4,3-*a*] pyrimidin-5(1*H*)-one 5a, B

A mixture of 2a (10 mmol, 1.4 g) or 2b (10 mmol, 1.68 g) and ammonium thiocyanate (30 mmol, 2.38 g) in glacial acetic acid (10 mL) was heated under reflux for 12 h. After cooling the reaction mixture was poured onto ice cold water. The precipitate formed was collected by filtration, washed with water, dried and crystallized from acetic acid to give the corresponding 5a, b in good yields.

#### 3-Amino-6-methyl- [1,2,4] triazolo[4,3-a] pyrimidin-5(*1 H*)-one (5a)

Yield (1.2 g, 74%), solid yellowish crystal m.p. 215–218 ºC., R_*f*_ = 0.25 (5% MeOH/CH_2_Cl_2_); IR (KBr, ν cm^− 1^): 3477, 3397 (NH_2_) 3291 (NH), 3113 (aromatic CH stretch.), 2994, 2921, 2774 (aliphatic CH, stretch.), 1622, (CONH), 1576 (C = N), 1528, 1466 (C = C ring stretch.); ^1^H NMR (500 MHz, DMSO-*d6*), δ (ppm): 1.82 (s, 3 H, CH_3_), 3.27–3.55 (br, 2 H, NH_2_) 7.10 (br,1H, NH) 7.18, (s, 1H, *H*_*7*_); ^13^C NMR (125 MHz, DMSO-*d6*), δ (ppm): 7.4, 98.2, 128.7, 145.0, 159.9, 166.1; MS (EI): *m*/*z* = 165.01 [M^+^], anal. calcd. for C_6_H_7_N_5_O (165.15): C, 43.63; H, 4.27; N, 42.4. Found C, 43.69; H, 4.32; N, 42.49%.

#### 3-Amino-7-propyl- [1,2,4] triazolo[4,3-*a*] pyrimidin-5(1*H*)-one (5b)

Yield (1.5 g, 78%), solid yellowish solid m.p. 66–68 ºC, R_*f*_ = 0.42 (5% MeOH/CH_2_Cl_2_); IR (KBr, ν cm^− 1^): 3406,3397 (NH_2_) 3292 (NH), 3012 (aromatic CH stretch.), 2962, 2873, (aliphatic CH, stretch.), 1639, (CONH), 1550 (C = N), 1502, 1409 (C = C ring stretch.); ^1^H NMR (500 MHz, CDCl_3_), δ (ppm): 1.00 (t, 3 H, CH_3,_*J* = 7.5 Hz), 1.75–1.79 (m, 2 H, CH_2_), 2.59 (t, 2 H, CH_2,_*J* = 7.5 Hz), 3.76–3.84 (br, 2 H, NH_2_, D_2_O exchangeable), 5.60 (s, 1H, *H*_*6*_), 7.33 (br,1H, NH, D_2_O exchangeable); ^13^C NMR (125 MHz, CDCl_3_), δ (ppm): 13.5, 21.5, 36.9, 97.0, 123.2, 143.8, 158.2, 171.0; MS (EI): *m*/*z* = 193 [M^+^], anal. calcd. for C_8_H_11_N_5_O (193.21): C, 49.73; H, 5.74; N, 36.25. Found C, 49.80; H, 5.78; N, 36.05%.

### Computational Methodology

The molecular modeling and photoelectronic properties of the studied compounds were investigated using Density functional theory (DFT) [34–43] methods via utilizing CAM-B3LYP [44] level with 6-311G++(d, p) [45] basis set. All computational calculations were presented via utilizing the Gaussian 16 program [46]. Upon utilizing DFT/CAM-B3LYP/6-31G++(d, p) level, the molecular structures (MSs) of neutral molecules are optimized in the gaseous phase, and their molecular electronic properties such as HOMO, LUMO levels, and the energy gap (E_g_) are obtained. The electrostatic potential and the calculated electronic absorption spectra in methanol for all studied MSs are calculated via applying the CAM-B3LYP/6-31G++(d, p) level of theory [47] using the Polarizable Continuum Model (PCM). The calculated electronic absorption spectra are calculated using time dependent density functional theory (TD-DFT) [48–51]. The total and partial density of states for all studied molecular structures are calculated using Multiwfn software [52].

### Molecular Docking

All docking studies and calculations were performed using AutoDock Vina, and Gasteiger partial charges were assigned during docking [53]. AutoDock is a suite of automated docking tools, which allows flexible ligand docking and is freely available under the GNU public license. The scoring function used is empirically derived, for empirical binding free energy force field that allows the prediction of binding free energy for docked ligands. The protein target needs to be prepared and modeled according to the format requirements of the docking algorithms used. Thus, the X-ray structure of DNA gyrase receptor PDB ID 4DUH was used. All bound water ligands were removed from the protein prior to the docking process.

The 2D chemical structures of the synthesized compounds and the co-crystallized ligand were sketched using ChemBioDraw Ultra 14.0. Polar hydrogen atoms were added, and Gasteiger partial charges were assigned to both the protein and ligands. The x, y, z AutoDock Vina grid center coordinates used were 18.62 Å,14.71 Å, 19.68 Å and the size of the search space was set to 20 Å x 20 Å x 20 Å and exhaustiveness = 8. The protein structure was subjected to energy minimization to optimize its conformation prior to docking. To evaluate the reliability of the docking protocol, molecular redocking of the co-crystallized ligand was performed. Validation was achieved by obtaining low RMSD values between the docked and experimentally determined (X-ray) structures. Docking of both the co-crystallized ligand and the synthesized compounds was then conducted using the default protocol parameters. Additionally, the binding energy estimation procedure included an energy minimization step consisting of 1,500 Steepest Descent steps followed by 500 Conjugate Gradient steps. The docking results obtained from the AutoDock program were further analyzed and visualized using PyMOL software to investigate the putative interaction mechanisms.

### Antimicrobial Activities Screening

The antimicrobial activity of synthesized compounds is sterilized by filtration using a 0.22 μm syringe filter and investigated against pathogenic Gram-positive bacteria represented by *Bacillus subtilis* ATCC6633 and *Staphylococcus aureus* ATCC6538, Gram-negative bacteria represented by *Pseudomonas aeruginosa* ATCC9022 and *Escherichia coli* ATCC8739. And unicellular fungi represented by *Candida albicans* ATCC10231 by agar well diffusion method. Approximately 100 µL of each bacterial and unicellular fungal strain was seeded individually on 20 mL Muller Hinton agar media and poured onto sterilized Petri dishes under aseptic conditions. After solidification, wells (7 mm in diameter) were made into seeded Petri dishes and filled with 100 µL of high concentration (100 µg mL^− 1^) of synthesized compounds dissolved in DMSO. To detect the minimum concentration from each compound that inhibits microbial growth, different concentrations (50, 25, 12.5 µg mL^− 1^) were prepared. DMSO was running with the experiment as a negative control, whereas ciprofloxacin as antibacterial and fluconazole as anti-candida are dissolved in DMSO at the same concentration and running with the experiment as a positive control. The results were recorded as a diameter of zone of inhibition around each well [54]. The experiment was done in triplicate. The ZOI was expressed as the mean ± standard deviation of three independent replicates. Data were subjected to analysis of variance (ANOVA) by the statistical package SPSS v17 (IBM, Armonk, NY, USA). The mean difference comparison between the treatments was analyzed by the Tukey HSD test at *p* < 0.05.

## Results and Discussion

### Synthesis

Many heterocyclic compounds can be synthesized using hydrazinopyrimidines derivatives from readily accessible starting materials such as 1,2,4-triazolo[4,3-a] pyrimidine derivatives. Although the title compounds, 1,2,4-triazolo[4,3-a]pyrimidin-5-(1 H)-one derivatives, have not yet been reported, but the synthetic methods of their preparations of [1,2,4]triazolo[4,3-a]pyrimidinone derivatives from 2-hydrazinopyrimidines have been reported in good yield [55, 56].

The treatment of 2-thioxo-2,3-dihydropyrimidin-4(*1 H*)-one derivatives 1a, b with hydrazine hydrate 99% in absolute ethanol under refluxed temperature afforded 2-Hydrazino-5-methylpyrimidin-4(3*H*)-one (2a) and 2-Hydrazinyl-6-propylpyrimidin-4(*3 H*)-one (2b). The reaction of key intermediates 2a, b with glacial acetic, triethyl orthoformate and ammonium thiocyanate, resulted in a cyclization reaction giving the corresponding 6- or 7-disustituted 3-methyl- [1,2,4]triazolo[4,3-*a*]pyrimidin-5(*1 H*)-one 3a, b [1,2,4], triazolo[4,3-*a*]pyrimidin-5(*1 H*)-one 4a, b and 3-amino [1,2,4]triazolo[4,3-*a*]pyrimidin-5(*1 H*)-one 5a, b, respectively (Fig. S1, Supplementary file).

The structure of the synthesized compounds was confirmed by elemental and spectral analyses: IR, ^1^H NMR, ^13^C NMR and by mass spectra (cf. experimental section. All the synthesized compounds were assessed for their in vitro antibacterial activity (against two Gram positive- and two Gram negative bacteria) and for their antifungal activity against Candida albicans.

### DFT Calculations

#### Optimized Molecular Structures

**Fig. 3:**
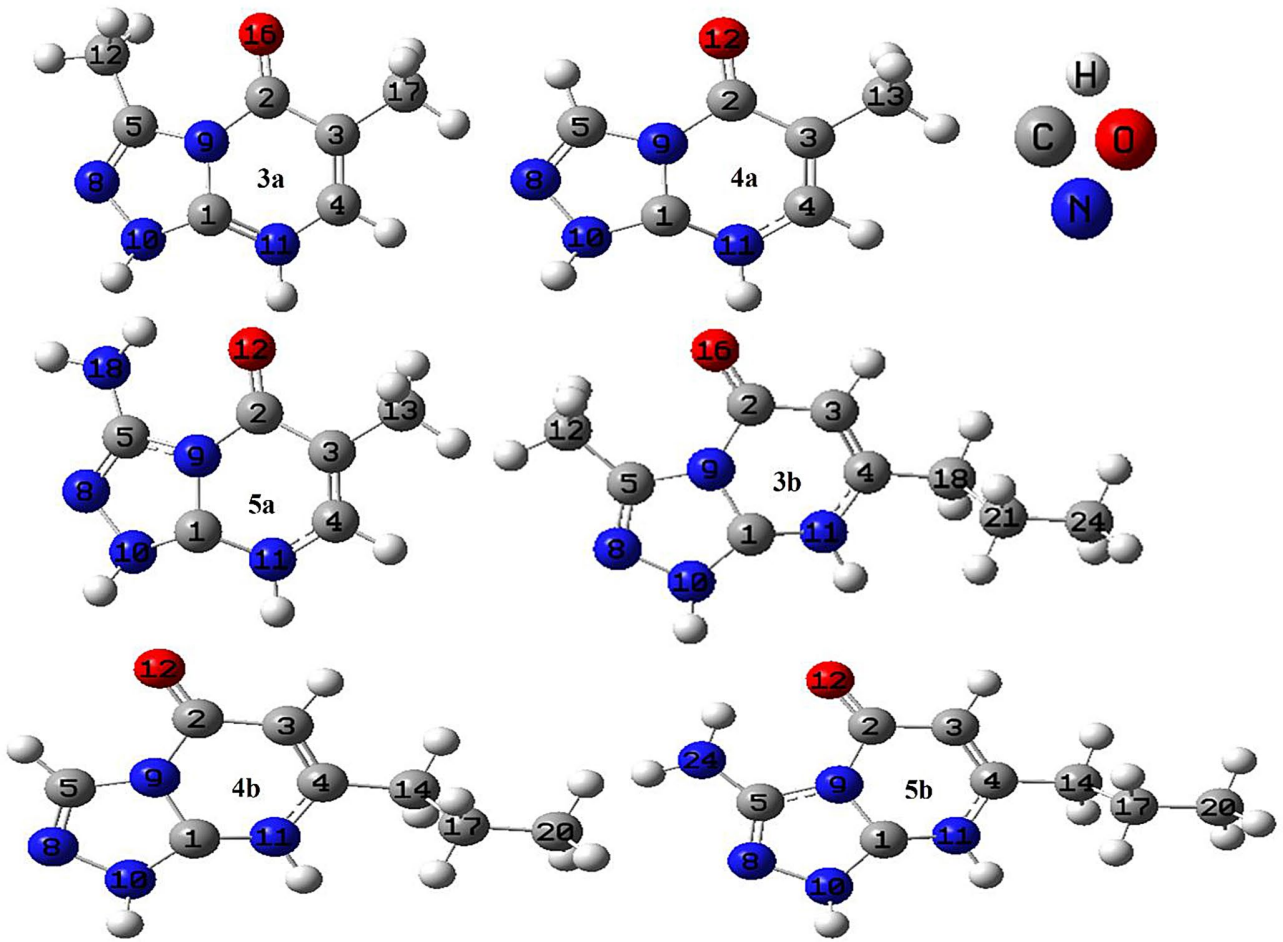
Optimized structures of **3a**, **5a**, **3b**, **4b**, and **5b** in gaseous phase

The optimized molecular structures (MS) of [1,2,4] triazolo[4,3-a] pyrimidin-5(1 H)-one (TPO) derivatives in the gaseous state are shown in Fig. 3. Table 1 presents the influence of substituents, such as methyl (CH_3_), amino (NH_2_), and propyl (C_3_H_7_), on important parameters of the optimized MS, including bond lengths (in Å), bond angles, and dihedral angles (in degrees) for molecules 3a, 4a, 5a, 3b, 4b, and 5b. The following comments can be deduced from the results: (i) Based on the listed dihedral angles, all the studied MSs exhibit a planar conformation. (ii) The dihedral angle C5-N9-C1-N10 consistently falls within the range of 10.689 to 11.647 degrees across all molecular structures (MSs). This observation suggests that the triazole ring is not coplanar with the pyrimidine ring. (iii) The dihedral angle C12-C5-N8-N10 is 176.476 degrees for 3a and 176.474 degrees for 3b, suggesting that the methyl (CH_3_) substituent lies in the same plane as TPO. (iv) The dihedral angle N18-C5-N8-N10 in molecule 5a is 173.676 degrees, indicating that the amino (NH_2_) substituent also lies in the same plane as TPO. (v) The dihedral angle C14-C4-N11-C1 is 164.164 degrees for 4b and 164.815 degrees for 5b, indicating that the propyl (C_3_H_7_) group lies in the same plane as TPO. (vi) Due to the increased bond order, the C5-N8 and C3-C4 bond lengths are shorter than the C1-N9 and C2-C3 bond lengths in all studied MSs. (vii) Based on the listed bond angle values, the hybridization type of the atoms in all studied MSs is sp². (viii) The C5-N8 and C3-C4 bond lengths in 3a, 5a, 3b, 4b, and 5b are longer compared to 4a. This can be attributed to the electron-donating properties of the CH_3_, NH_2_, or C_3_H_7_ substituents.

To validate our computational results, we compared key optimized structural parameters (bond lengths and angles) of our 1,2,4-triazolo[4,3-a] pyrimidin-5(1 H)-one derivatives with data from related triazolopyrimidine systems. Our compounds are based on the [4,3-a] fusion isomer, while M. Abul Haja et al. reported values for 5-oxo and 7-oxo derivatives of the [1,2,4]triazolo[1,5-a]pyrimidine isomer [57]. Due to differing fusion patterns, direct atom-to-atom comparisons are not applicable. Therefore, we focused on analogous bond types and geometric features. Our calculated bond lengths for compounds 3a, 4a, 5a, 3b, 4b, and 5b are: C5–N8 (1.277–1.285 Å), C3–C4 (1.353–1.360 Å), C1–N9 (1.411–1.416 Å), and C2–C3 (1.446–1.463 Å). In comparison, the [1,5-a] isomer values reported are: C5–N8 (1.380 Å), C3–C4 (1.454 Å), C1–N9 (1.356 Å), and C2–C3 (1.338 Å). For bond angles, our values are: N9–C5–N8 (111.591–112.888°) and C1–N9–C2 (122.91–124.87°), compared to 122.9° reported for the [1,5-a] isomer. Despite structural differences, the bond lengths and angles in our compounds are consistent with those of related heterocyclic systems, aligning with expected values based on hybridization and electronic effects. These results support the accuracy of our computational methodology.

**Table 1:** Selected optimized structural parameters (bond length in ˚A, bond angle, and dihedral angle in degree computed for **3a**, **5a**, **3b**, **4b**, and **5b** in gaseous phase. For labeling, Fig. 3

Designation	3a	4a	5a	3b	4b	5b	[57]
C5-N8	1.281	1.277	1.285	1.281	1.278	1.284	1.380
C3-C4	1.354	1.353	1.356	1.358	1.359	1.360	1.454
C1-N9	1.416	1.414	1.415	1.414	1.411	1.415	1.356
C2-C3	1.463	1.461	1.458	1.451	1.449	1.446	1.338
N9-C5-N8	111.591	112.818	112.611	111.661	112.888	112.713	-
C1-N9-C2	123.308	124.87	123.91	122.91	124.426	124.116	122.9
N10-C1-N11	120.961	121.528	121.396	120.663	121.225	120.554	-
C5-N9-C1-N10	11.600	11.033	10.689	11.422	10.769	11.647	-
C12-C5-N8-N10	176.476	-	-	176.474	-	-	-
N18-C5-N8-N10	-	-	173.676	-	-	-	-
C13-C3-C4-N11	-	178.921	179.898	-	-	-	-
C14-C4-N11-C1		-	-	-	164.164	164.815	-

#### Molecular Structures Stability

**Fig. 4:**
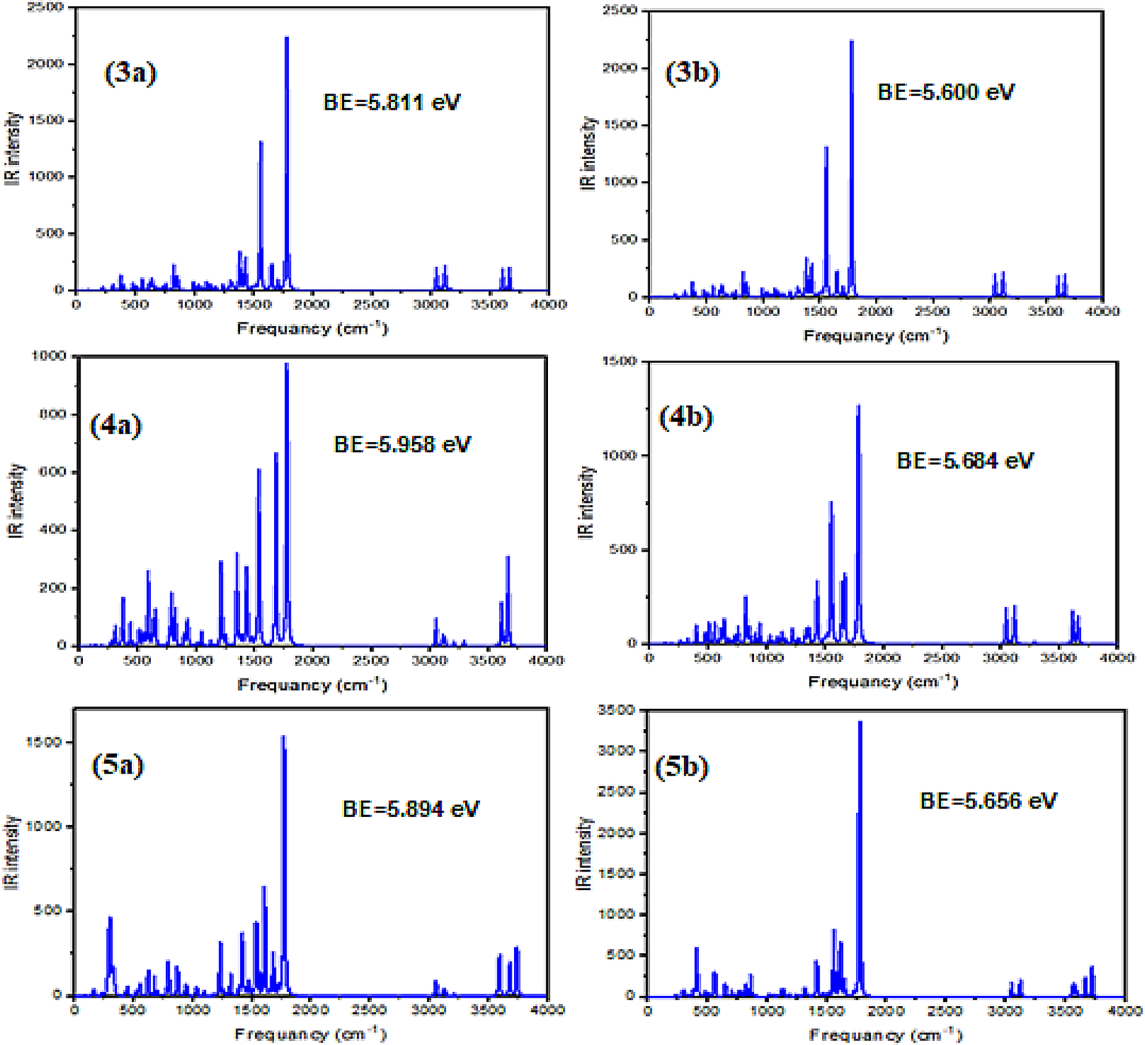
Calculated IR and BE of **3a**,** 3b**,** 4a**,** 4b**,** 5a and 5b** MSs in gaseous state

The stability of the following MSs: 3a,4a,5a,3b,4b, and 5b are investigated by calculating the binding energy (BE) and by performing frequency calculations. The BE is calculated from the equation; BE = (N_C_E_C_+ N_H_E_H_+N_N_E_N_+N_O_E_O_-E_t_)/N_t_. With N_C_, N_H_, N_N_, N_O_ and N_t_ are the numbers of C, H, N, O, and the total number of atoms, respectively. E_C_, E_H_, E_N_, E_O_, and E_t_ are the corresponding total energies of the C, H, N, O and the final compound, respectively. We analyzed the binding energies and infrared (IR) spectra to evaluate the stability and characteristics of TPO and its derivative compounds. The calculated binding energies, ranging from 5.600 to 5.958 eV, consistently indicate the stability of the molecular structures (MSs) of TPO and its derivatives. These values have been illustrated in Fig. 4 for each structure. Notably, certain modifications made to TPO affected the binding energy. Specifically, compounds 3a, 5a, 3b, 4b, and 5b exhibited lower binding energies compared to compound 4a. This suggests that the addition of methyl, amino, and propyl groups enhances the reactivity of the studied MSs due to their electron-donating properties. To further assess the dynamic stability of the structures, we analyzed the IR spectra obtained from frequency calculations. The positive vibrational frequencies presented in Fig. 4 indicate the absence of saddle points on the potential energy surface, confirming the dynamic stability of all considered structures. Additionally, we propose that the IR spectra can serve as a useful tool for identifying TPO and its derivatives in experimental synthesis. By comparing the experimental and computational IR spectra, we can observe notable differences. For example, compound 4a in the experimental synthesis exhibits vibrational IR peaks (ν cm^-1^) at 3255 (NH), 3097 (aromatic CH stretch), 2940, 2859 (aliphatic CH stretch), 1691 (CONH), 1608 (C = N), 1526, 1470 (C = C ring stretch) (See Sect. 6-Methyl- [1,2,4] triazolo[4,3-*a*] pyrimidin-5(1*H*)-one (4a) and supplementary file). Conversely, the computational IR spectra for the same compound exhibit vibrational IR peaks (ν cm^‐1^) at 3673.07 (NH), 3131.24 (aromatic CH stretch), 3054 (aliphatic CH stretch), 1779.80 (CONH), 1687.02 (C = N), 1539.27, 1433.96 (C = C ring stretch). Note that the calculated frequencies are slightly higher than the observed values for most normal modes. Two factors may account for the discrepancies between the experimental and computed spectra of TPO MSs. Firstly, these discrepancies could be caused by the environmental conditions during the experimental measurements. Secondly, the experimental value represents an anharmonic frequency, while the calculated value corresponds to a harmonic frequency [58].

#### Density of State (DOS) and HOMO/LUMO MOs

**Fig. 5:**
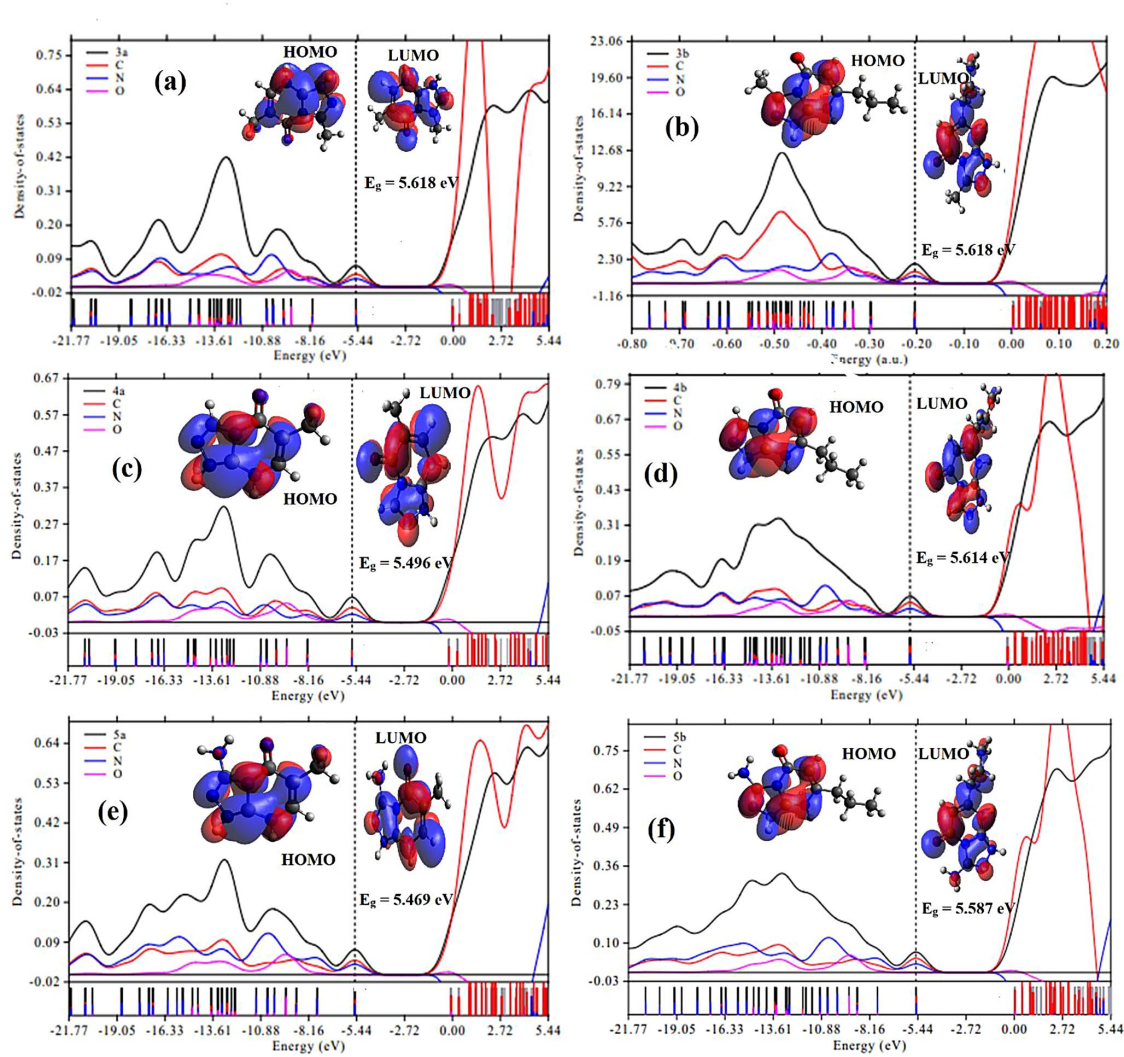
Total and partial density of states of **3a**, **5a**, **3b**, **4b**, and **5b** (**a**-**f**) and the corresponding HOMO/LUMO graps (**a**-**f**)

To study the electronic properties, the partial density of states (PDOS) and the highest occupied/lowest unoccupied molecular orbitals (HOMO/LUMO) are employed. The PDOS and (HOMO/LUMO) shown in Fig. 5 (a-f) were obtained by further analysis of the Gaussian output file using the Multiwfn software that calculates the percentage contribution of each atom to the molecular orbitals. The overlapping of PDOS spectra and the observation of C peaks being higher than N and O peaks in the PDOS of all studied compounds (See Fig. 5 (a-f)) can be attributed to several factors related to the electronic structure and bonding properties of the molecule. Here are a few possible explanations for these results: (i) Hybridization and Molecular Orbitals: The overlapping PDOS spectra suggests the presence of strong orbital interactions and delocalization of electrons within the molecule. The C, N, and O atoms in the molecule likely undergo hybridization, resulting in the formation of molecular orbitals that extend over multiple atoms. This electron delocalization can lead to overlapping PDOS peaks. (ii) Conjugation and Resonance: The molecular structures of 3a, 5a, 3b, 4b, and 5b may possess conjugated systems or resonance structures, which allow for electron delocalization across the molecule. This can contribute to the overlapping PDOS spectra and potentially result in enhanced electron densities on the carbon atoms. (iii) Electronic Effects: The electronic properties of C, N, and O atoms, such as electronegativity and atomic size, can influence their PDOS spectra. The higher PDOS peaks observed for carbon compared to nitrogen and oxygen might be attributed to differences in the electronegativity or orbital energies of these atoms. Additionally, the presence of functional groups or substituents in the molecule can affect the electron distribution and thus influence the relative intensities of the PDOS peaks. The observed Fig. 3 (a-f) in the PDOS shows that in the HOMO (highest occupied molecular orbital), there are multiple DOS peaks associated with oxygen, nitrogen, and carbon atoms. However, in the LUMO (lowest unoccupied molecular orbital), there is only a single peak for the carbon atom, and the peaks for nitrogen and oxygen atoms are absent. The reason for this difference in DOS peaks can be attributed to the electronic structure and bonding characteristics of the atoms involved. In the HOMO, where the electrons are primarily localized, the oxygen, nitrogen, and carbon atoms contribute to multiple DOS peaks due to their different electronic configurations and interactions with neighboring atoms. In contrast, in the LUMO, which represents the lowest energy level available for electron acceptance, the presence of a single DOS peak for the carbon atom suggests that it has the highest affinity for accepting electrons among the atoms in the molecule. The absence of DOS peaks for nitrogen and oxygen atoms in LUMO indicates a lower affinity for accepting electrons than carbon.

The observations made regarding the localization of the highest occupied molecular orbital (HOMO) and lowest unoccupied molecular orbital (LUMO) on the triazole and pyrimidine rings, as well as the contributions of substituent groups, can provide insights into the electronic structure and reactivity of the compounds. Here’s a discussion and possible explanations for these findings: (i) Localization of HOMO and LUMO: The localization of the HOMO and LUMO on the triazole and pyrimidine rings (See Fig. 5 (a-f)) suggests that these regions of the molecule are involved in the highest and lowest energy electron densities, respectively. This localization could indicate that these rings play a significant role in the electronic and chemical properties of the compounds. (ii) Substituent effects on HOMO and LUMO: The contributions of substituent groups to HOMO and LUMO can be influenced by their electronic and steric effects. In compounds 3b, 4b, and 5b, the propyl substituent groups contribute to the LUMO but not the HOMO (See Fig. 5 (b, d, and f). This observation suggests that the propyl groups may have an impact on the electron-accepting behavior or the reactivity of these compounds. In compounds 4a and 5a, the methyl substituent groups contribute to the HOMO but not the LUMO (See Fig. 5 (c and e), indicating that these groups are involved in the electron-donating behavior of the compounds. The small contribution in the HOMO suggests that the presence of the methyl groups has a modest effect on the electron density distribution. In compound 3a, the methyl group does not contribute to either the HOMO or the LUMO as shown in Fig. 5a. This result suggests that the methyl group has minimal influence on the electronic structure and reactivity of the compound. In compound 5a, there is a small contribution from the NH_2_ group in the HOMO, indicating its involvement in the electron-donating behavior. However, it does not contribute to the LUMO, implying a different electronic interaction or reactivity in this region. The observed localization of HOMO and LUMO on the triazole and pyrimidine rings aligns with the PDOS results, where the carbon peaks were higher than the nitrogen and oxygen peaks. This suggests that the carbon atoms in these rings have a more significant influence on the overall electronic density and bonding properties in the compounds.

#### Quantum Stability

Important quantum stability chemical (QSC) parameters like dipole moment (µ), chemical potential (ρ), electronegativity (χ), and chemical hardness (η) were calculated using the HOMO energy (E_H_) and the LUMO (E_L_). These QSC parameters are obtained from the following equations $\:{\uprho\:}=\frac{{E}_{H}+\:{E}_{L}}{2}\:$, χ $\:=\:-\frac{{E}_{H}+{E}_{L}}{2},\:\text{a}\text{n}\text{d}\:{\upeta\:}=\frac{{E}_{L}-{E}_{H}}{2}$ [41] and collected in Table 2. The dipole moment (µ) reflects the asymmetry in the electronic charge distribution of a structure. Among the structures examined, 4b exhibited the highest magnitude of µ, indicating a greater extent of active intramolecular charge transfer compared to other structures. Following 4b, 3b had the second highest dipole moment. The chemical potential (ρ) measures the tendency of electrons to escape from a structure. In this case, 4a had the most negative ρ value compared to the other compounds, suggesting a higher propensity for electron escape. Electronegativity (χ) is a measure of an atom or structure’s ability to attract electrons. 4a exhibited the highest electronegativity values, indicating a strong tendency to attract electrons. The electronegativity values for 4b were slightly lower, while the presence of NH_2_ and CH_3_ substituents decreased the electronegativity to 2.731. Chemical hardness (η) represents the resistance to charge transfer. Among the compounds, 3b had the highest hardness, indicating a greater resistance to charge transfer. On the other hand, 5a had the lowest hardness, implying lower resistance to charge transfer. Based on these observations, it can be concluded that the reactivity increases when electron-donating substituents like NH_2_ and CH_3_ are added to the compounds. The presence of these substituents leads to a decrease in electronegativity and an increase in reactivity.

**Table 2:** The HOMO energy (E_H_), the LUMO energy (E_L_), chemical potential (ρ), electronegativity (χ), chemical hardness (η), and dipole moment (µ) for selected **3a**, **5a**, **3b**, **4b**, and **5b**

Compound	H	L	ρ(eV)	χ(eV)	η(eV)	µ (D)
3a	-5.555	-0.037	-2.796	2.796	2.759	4.160
3b	-5.54	0.078	-2.731	2.731	2.809	5.255
4a	-5.683	-0.187	-2.935	2.935	2.748	5.011
4b	-5.676	-0.062	-2.869	2.869	2.807	6.133
5a	-5.544	-0.075	-2.810	2.810	2.735	3.496
5b	-5.549	0.038	-2.756	2.756	2.794	4.838

### Molecular Electrostatic Potentials

**Fig. 6:**
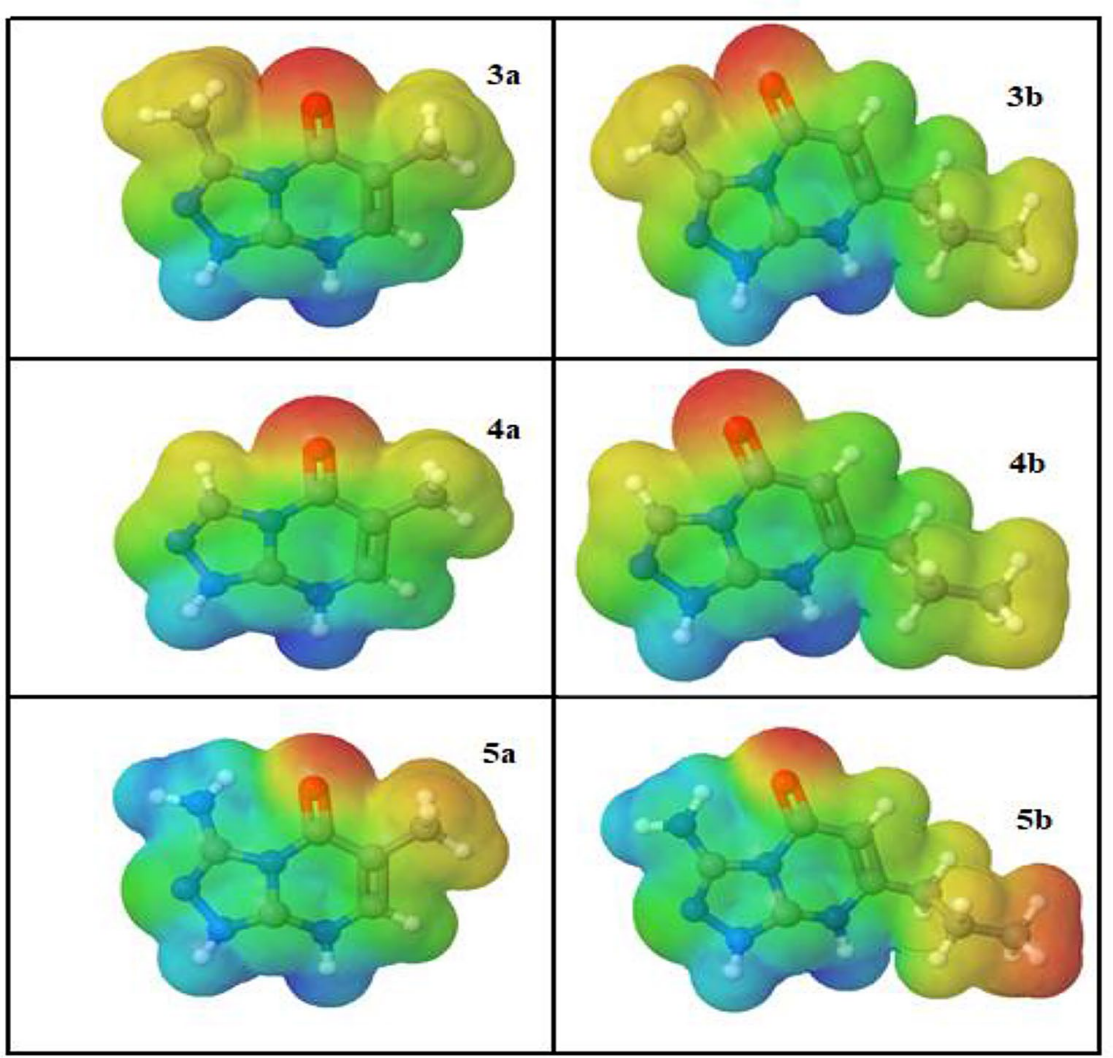
Electrostatic potential map (ESP) for **3a**, **5a**, **3b**, **4b**, and **5b** molecular structures

The molecular electrostatic potential (MEP) analysis was conducted to identify the electrophilic and nucleophilic sites in 3a, 3b, 4a, 4b, 5a, and 5b [59]. Figure 6 displays the MEPs, with a color scheme representing different charge densities. The color scheme is as follows: red for regions with an electron-rich, partially negative charge; blue for regions with an electron-deficient, partially positive charge; light blue for slightly electron-deficient regions; yellow for slightly electron-rich regions, and green for neutral (zero potential) regions (refer to Fig. 6). The carbonyl group is surrounded by a negative region (red color) in the MEP surface, indicating that this area is electron-rich and susceptible to electrophilic attacks. On the other hand, the hydrogen atoms linked to nitrogen atoms (N-H) exhibit a positive region (blue color) in the MEP, suggesting that these sites are electron-deficient and prone to nucleophilic attack. Therefore, based on the MEP analysis, the carbonyl group represents an electrophilic site, while the N-H bonds serve as nucleophilic sites in 3a, 3b, 4a, 4b, 5a, and 5b. This information is valuable for predicting potential reaction sites and understanding the reactivity of these compounds.

### UV-Vis Spectra

**Fig. 7:**
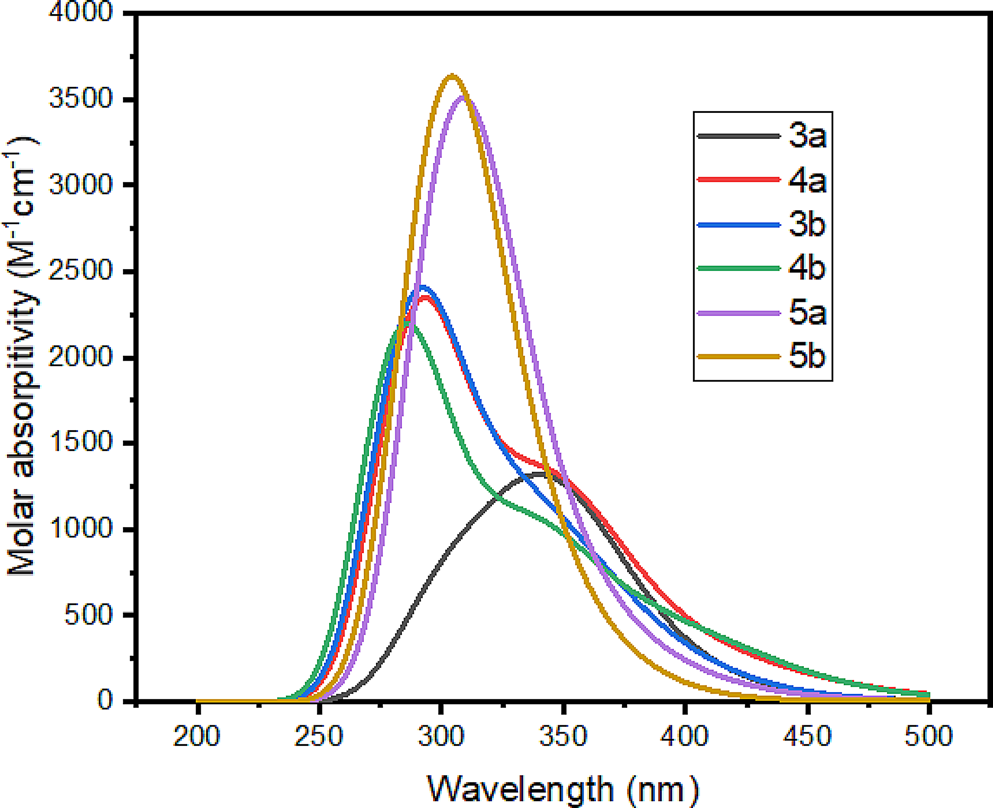
The calculated electronic absorption spectra for **3a**, **5a**, **3b**, **4b**, and **5b** molecular structures in methanol

In previous studies [60–62], the absorption spectra of triazolo-pyrimidine materials were investigated within the UV-region range of 250–400 nm. These spectra were attributed to the π-π* electronic transitions. The CAM-B3LYP functional was employed to accurately calculate the UV-Vis absorption spectra. The results, depicted in Fig. 7 and summarized in Table 3, showed that the computational absorption spectra for all the studied materials (MSs) fell within the 250–400 nm range, consistent with the experimental data. A comparison between the computational and experimental results revealed a good agreement, further validating the computational approach. The maximum calculated electronic absorption wavelengths for 3a, 4a, and 5a were found to be 343.41, 297.35, and 306.29 nm, respectively, corresponding to H→L, H→L + 2, and H-1→L electronic transitions. Similarly, for 3b, 4b, and 5b, the maximum calculated absorption wavelengths were 296.52, 288.08, and 303.46 nm, respectively, due to H→L + 3, H→L + 2, and H→L + 2 electronic transitions. Notably, the maximum absorption spectra of 3a and 5a exhibited a redshift compared to 4a, whereas the maximum absorption spectra of 3b and 5b showed a redshift compared to 4b. This redshift can be attributed to the electron-donating properties of the CH_3_ and NH_2_ substituents. Additionally, the presence of the propyl group substituent caused the maximum absorption spectra of 3a, 4a, and 5a to be redshifted compared to the maximum absorption spectra of 3b, 4b, and 5b, respectively (as illustrated in Fig. 7; Table 3). In conclusion, the computational results successfully reproduced the experimental UV-Vis absorption spectra of the triazolo-pyrimidine materials. The observed shifts in the absorption maxima for different substituents provide insights into the electronic properties of the studied compounds, highlighting the influence of specific groups on the absorption characteristics within the UV range.

**Table 3:** The calculated excited state (ES), maximum wavelength (λ_max_), transition energy (TE), electronic transition (ET), oscillator strength (*f*), and transition coefficient (TC)

Compounds	ES	λ_max_	TE (eV)	ET	f	TC
3a	4	343.41	3.610	H→L	0.019	0.1292
3b	5	296.52	4.181	H→L + 3	0.041	0.6245
4a	5	297.35	4.170	H→L + 2	0.046	0.7767
4b	5	288.08	4.304	H→L + 2	0.033	0.4380
5a	5	306.29	4.048	H-1→L	0.051	0.1706
5b	5	303.46	4.086	H→L + 2	0.068	0.1680

### Molecular Docking

**Fig. 8:**
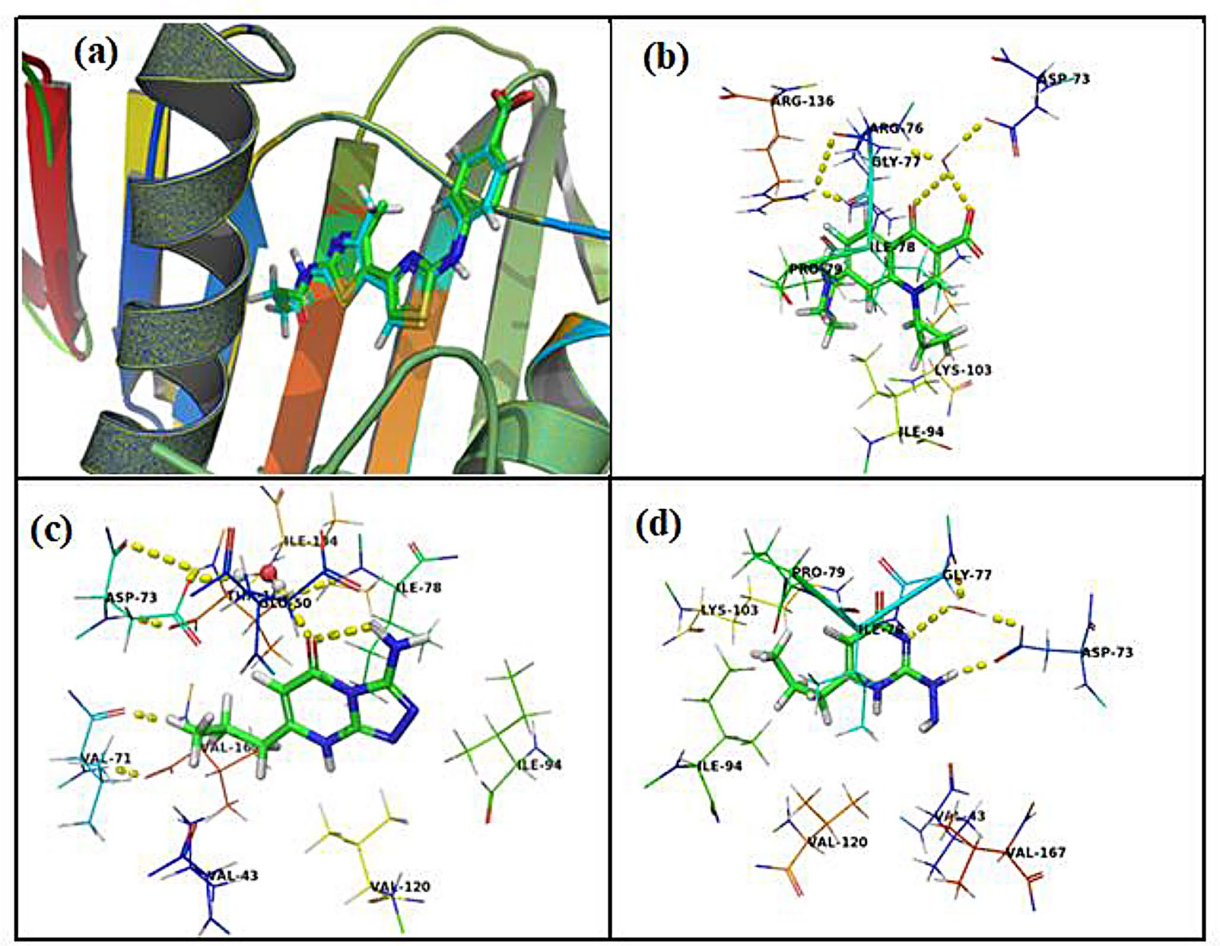
Predicted binding modes with the DNA gyrase receptor: (**a**) co-crystallized **ligand**, (**b**) **ciprofloxacin**, (**c**) compound **5b**, and (**d**) compound **2b**. Interactions between hydrogen-bonded atoms are indicated by yellow dotted lines. Atom color coding: hydrogen (white), nitrogen (blue), oxygen (red), and sulfur (yellow)

To assess the antimicrobial potential of the synthesized compounds, their binding modes were investigated through molecular docking studies as shown in Fig. 8 (a-d). The AutoDock program [53] was employed for this purpose, using the DNA gyrase receptor with PDB ID 4DUH as the target. To ensure the accuracy of the docking procedure in predicting the binding conformations, redocking experiments were performed for reference ligands, namely ciprofloxacin and essramycin [63]. The results of these redocking experiments served as a validation for the reliability of the docking methodology. The outcomes of the docking study were reported as the binding free energy of binding (∆G), as shown in Table 4. The results indicated that all the target compounds exhibited similar positions and orientations within the putative binding site of the DNA gyrase receptor. This suggests that the synthesized compounds have the potential to interact with the receptor and exert antimicrobial effects through their binding interactions.

**Table 4:** The Docking binding free energies of the synthesized compounds with DNA gyrase receptor

Compound	ΔG kcal/mol
Co-crystallized ligand	-90.48
ciprofloxacin	-70.28
Essramycin	-60.56
**2a**	-50.42
**2b**	-50.85
**3a**	-50.27
**3b**	-50.22
**4a**	-50.61
**4b**	-40.84
**5a**	-50.53
**5b**	-60.47

The proposed binding mode of redocking of co-crystallized ligand indicated the same position and orientation inside the binding site keeping the same hydrophilic and hydrophobic interactions with free energy of binding value of -90.48 kcal/mol **(**Fig. 8a). The binding mode results for ciprofloxacin with DNA gyrase receptor follow the general pattern observed for co-crystallized ligand and showed affinity value of -70.28 kcal/mol. The carboxylate and carbonyl groups of ciprofloxacin were involved in water mediated interactions with Asp73 and the amino group of Glu77. The bicyclic core of ciprofloxacin was in hydrophobic interactions with Ile78 and Pro79. The propyl moiety was stabilized by hydrophobic interactions with Ile94 and hydrophobic part of Lys103. Piperazine moiety can increase and accommodate the affinity towards DNA gyrase receptor through the hydrophilic interactions with hydrophobic parts of Arg76 and Arg136 (Fig. 8b).

The obtained binding mode results of 5b with DNA gyrase receptor allowed us to propose that the bicyclic core of 5b is stabilized by aliphatic hydrophobic interactions with Val43, Thr165, Ile78, Ile94, and Val120 Val250 and the carbonyl group at the 5-position is formed a water mediated interactions Asp73, backbone carbonyl group of Ile134 and Thr165 Asn254 (Fig. 6c). The amino group at the 3-position of compound 5b is at a distance of 3.9 from Glu50 which stabilized compound 5b inside the DNA gyrase receptor. In addition, the propyl moiety is located inside the hydrophobic interactions formed by Val43, Val71, and Val167. Synthesized compound 5b has a nice capacity to accommodate the putative binding site and therefore, displayed the highest affinity towards the DNA gyrase receptor compared with other active synthesized compounds.

However, the binding mode for compound 2b showed affinity value of -50.85 kcal/mol can decrease the affinity for the DNA gyrase receptor compared with compound 5b, displayed affinity value of -60.47 kcal/mol where the differences between the affinities of compounds 2b and 5b could be explained by unfavorable interactions between the compound 2b and the DNA gyrase receptor e.g. the hydrazine moiety was surrounded by unfavorable interactions with nonpolar groups like, Ala47, Val43, and Val120. In detail, the results of docking analysis of the synthesized compound 2b with DNA gyrase receptor displayed that hydrazine moiety was involved in a salt bridge with Asp73 and the hydrogen bonding interaction with the backbone amino group of Gly77. Moreover, the propyl moiety was in hydrophobic interactions formed by Ile78, Pro79, Ile94 and the hydrophobic part of Lys103, a common binding mode for the synthesized derivatives (Fig. 6d). Moreover, the structural findings of the other synthesized compounds were accompanied by energetic aspects the observed binding energies ΔG for each complex are listed. The experimentally measured values ranged from − 10.74 to -60.47 kcal∙mol. As shown in (Table 4, the computed values reflect the overall trend.

### Antimicrobial Activity

The antibacterial and antifungal activities of the target compounds have been investigated by measuring their inhibitory effect against two Gram-positive strains namely *Bacillus subtilis* and *Staphylococcus aureus*, two Gram-negative bacteria including *Pseudomonas aeruginosa* and *Escherichia coli* and a fungus namely *Candida albicans* using agar diffusion technique [64, 65]. Ciprofloxacin and fluconazole were used as a positive control drugs [54]. The observed inhibition zone of the compounds and the reference drugs are given in Tables 5, 6 and 7. DMSO (negative control) did not exhibit any antimicrobial activity. On the other hand, the antimicrobial activity results revealed that the tested compounds exhibited various degrees of inhibition against the microorganisms used. Data analysis showed that the activity of positive control against *B. subtilis*, *S. aureus*, and *E. coli* is significant (*p* ˂ 0.001) as compared to synthesized compounds. The highest concentration (100 µg mL^− 1^) of ciprofloxacin forming zone of inhibitions (ZOIs) of 27.3 ± 0.6, 23.7 ± 1.2, and 22.7 ± 0.6 mm for *B. subtilis*, *S. aureus*, and *E. coli* whereas the highest ZOIs formed due to synthesized compounds were 22.3 ± 0.6 mm (*B. subtilis)*, 20.7 ± 0.6 mm (*S. aureus)*, and 16.3 ± 0.6 mm (*E. coli).*

The most active compounds for Gram-positive bacteria were compounds 5b, 2b, and 5a, respectively, which showed inhibition zones against one or more of the tested Gram-positive bacteria. On the other hand, the most active compounds for Gram-negative bacteria were compounds 2b, 5a, and 5b. However, fungi strains are considered the most sensitive among the tested microorganisms for all except compound 4a which behave as no effective compound.

On the other hand, the activity of compound 5b was significant (*p* ˂ 0.001) against *B. subtilis* and *C albicans* recording ZOIs of 22.3 ± 0.6 mm and 20.7 ± 0.6 mm respectively, as compared to ciprofloxacin (27.3 ± 0.6 mm) and fluconazole (17.3 ± 0.6 mm). The highest activity of 5b could be attributed to the presence of amino group at the 3-position and propyl moiety at the 7-position of triazolo-pyrimidine scaffold.

As well, the activity of compound 2b was significant (*p* ˂ 0.001) against *S. aureus* and *P aeruginosa* recording ZOIs of 20.7 ± 0.6 mm and 31.3 ± 1.2 mm respectively, as compared to ciprofloxacin recording (23.7 ± 1.2 mm) and (20.7 ± 1.2 mm) respectively. The highest activity of 2b could be attributed to the presence of hydrazine moiety at the 2-position and propyl moiety at the 6-position of pyrimidine scaffold. The inhibitory activity of these compounds seems to be more effective against bacterial strains than fungal ones. In addition, compound 2b showed excellent inhibitory profile recording ZOIs of 24.3 ± 0.6 mm and 24.3 ± 0.6 mm respectively, as compared to fluconazole recording (17.3 ± 0.6 mm) and appeared from substitution of triazolopyrimidine scaffold. Tables 5, 6 and 7.

The MIC value is a critical factor should be detected before incorporation of active compounds in medications [66]. Analysis of variance showed that the MIC values for *B. subtilis* were 100, 50, and 25 µg mL^–1^ for compounds (3a), (2b and 4b), and (5b) respectively (Table 5). On the other hand, the MIC values for synthesized compounds against *S aureus* were 100 and 25 µg mL^–1^ for (4a) and (2a, 2b,4b, 5a, and 5b) respectively (Table 5). Data analysis showed that the MIC values for Gram-negative bacteria were 100 µg mL^–1^ (4a, 4b, and 5b), 50 µg mL^–1^ (5a), and 25 µg mL^–1^ (2b) for *P. aeruginosa* and *E. coli*. Interestingly the synthesized compounds 2a, 3a, 3b showed activity against *E. coli* with MIC value of 50 µg mL^–1^ (Table 6). Except 4a compound, all synthesized compounds showed anti-*Candida* activity with MIC value of 100 µg mL^–1^ for 5a, 50 µg mL^–1^ for 4b, and 25 µg mL^–1^ for 2a, 2b, 3a, 3b, and 5b (Table 7). In a similar study, some pyrimidine derivatives showed antimicrobial activity against pathogenic Gram-positive, Gram-negative strains, and unicellular fungi with varied MIC values [67].

The Gram-negative bacteria P. aeruginosa was the highest sensitive to tested compound recording the highest ZOI, and this could be attributed to the cell wall structure between Gram-positive and gram-negative bacteria. The cell wall of Gram-negative bacteria is composed of a thin layer of peptidoglycan and rich in phospholipid layer, contrary to Gram-positive bacteria which is composed of a thick peptidoglycan layer. The synthesized compounds can easily penetrate the thin peptidoglycan layer and react with the cytoplasmic membrane ultimately hindering the selective permeability function [68, 69]. The activity of synthesized compounds against Candida sp. could be related to their activity to dysfunction of ergosterol pathway via disruption of sterol profile in their cell walls (Fouda et al. 2022).

**Table 5:** The activity of different concentrations of synthesized compounds against Gram-positive bacteria, *Bacillus subtilis*, *Staphylococcus aureus*. Data recorded as a zone of Inhibition (ZOI) by mm

Treatment	Concentrations (µg mL^–1^)/ ZOIs (mm)							
	Bacillus subtilis				Staphylococcus aureus			
	100	50	25	12.5	100	50	25	12.5
Ciprofloxacin (positive control)	27.3 ± 0.6^a^	22.0 ± 1.0^a^	14.7 ± 0.6^a^	9.7 ± 0.6^a^	23.7 ± 1.2^a^	19.7 ± 1.2^a^	13.7 ± 0.6^a^	9.0 ± 0.0^a^
**2a**	0.0 ± 0.0	0.0 ± 0.0	0.0 ± 0.0	0.0 ± 0.0	16.7 ± 1.6^c^	10.6 ± 0.6^c^	8.7 ± 0.6^b^	0.0 ± 0.0^b^
**2b**	14.3 ± 0.5^c^	10.3 ± 0.5^cd^	0.0 ± 0.0	0.0 ± 0.0	20.7 ± 0.6^b^	16.7 ± 0.6^b^	11.7 ± 1.2^ab^	0.0 ± 0.0^b^
**3a**	10.3 ± 0.5^d^	0.0 ± 0.0^e^	0.0 ± 0.0	0.0 ± 0.0	0.0 ± 0.0	0.0 ± 0.0	0.0 ± 0.0	0.0 ± 0.0^b^
**3b**	0.0 ± 0.0	0.0 ± 0.0	0.0 ± 0.0	0.0 ± 0.0	0.0 ± 0.0	0.0 ± 0.0	0.0 ± 0.0	0.0 ± 0.0^b^
**4a**	0.0 ± 0.0	0.0 ± 0.0	0.0 ± 0.0	0.0 ± 0.0	15.0 ± 0.0^c^	0.0 ± 0.0	0.0 ± 0.0	0.0 ± 0.0^b^
**4b**	15.3 ± 0.6^c^	12.3 ± 0.6^cd^	0.0 ± 0.0	0.0 ± 0.0	15.3 ± 0.6^c^	14.3 ± 0.6^b^	11.3 ± 0.6^ab^	0.0 ± 0.0^b^
**5a**	0.0 ± 0.0	0.0 ± 0.0	0.0 ± 0.0	0.0 ± 0.0	19.0 ± 1.0^b^	15.0 ± 1.0^b^	11.3 ± 0.6^ab^	0.0 ± 0.0^b^

Values within the same column with different letters are significantly different (*p* ≤ 0.05), values are means ± SD (*n* = 3)

**Table 6:** The activity of different concentrations of synthesized compounds against Gram-negative bacteria, *Pseudomonas aeruginosa*,* Escherichia coli* data recorded as a zone of Inhibition (ZOI) by mm

Treatment	Concentrations (µg mL^–1^)/ ZOIs (mm)							
	Pseudomonas aeruginosa				Escherichia coli			
	100	50	25	12.5	100	50	25	12.5
Ciprofloxacin (positive control)	20.7 ± 1.2^b^	17.3 ± 0.6^b^	12.6 ± 1.2^b^	9.3 ± 0.6^a^	22.7 ± 0.6^a^	20.3 ± 0.6^a^	14.3 ± 0.6^a^	9.7 ± 0.6^a^
**2a**	0.0 ± 0.0^f^	0.0 ± 0.0^e^	0.0 ± 0.0^c^	0.0 ± 0.0^b^	11.7 ± 0.6^d^	9.7 ± 0.6^c^	0.0 ± 0.0^c^	0.0 ± 0.0^b^
**2b**	31.3 ± 1.2^a^	23.7 ± 1.2^a^	14.3 ± 0.6^a^	0.0 ± 0.0^b^	13.3 ± 1.5^cd^	11.7 ± 1.2^c^	9.3 ± 0.6^b^	0.0 ± 0.0^b^
**3a**	0.0 ± 0.0^f^	0.0 ± 0.0^e^	0.0 ± 0.0^c^	0.0 ± 0.0^b^	13.7 ± 1.6^cd^	11.7 ± 0.6^c^	0.0 ± 0.0^c^	0.0 ± 0.0^b^
**3b**	0.0 ± 0.0^f^	0.0 ± 0.0^e^	0.0 ± 0.0^c^	0.0 ± 0.0^b^	12.3 ± 0.6^d^	11.0 ± 1.0^c^	0.0 ± 0.0^c^	0.0 ± 0.0^b^
**4a**	15.7 ± 0.6^d^	0.0 ± 0.0^e^	0.0 ± 0.0^c^	0.0 ± 0.0^b^	10.3 ± 0.6^d^	0.0 ± 0.0^d^	0.0 ± 0.0^c^	0.0 ± 0.0^b^
**4b**	15.7 ± 1.2^d^	0.0 ± 0.0^e^	0.0 ± 0.0^c^	0.0 ± 0.0^b^	12.7 ± 0.6^d^	0.0 ± 0.0^d^	0.0 ± 0.0^c^	0.0 ± 0.0^b^
**5a**	18.7 ± 1.2^c^	14.7 ± 0.6^c^	0.0 ± 0.0^c^	0.0 ± 0.0^b^	14.7 ± 0.6^bc^	10.70.6^c^	0.0 ± 0.0^c^	0.0 ± 0.0^b^

Values within the same column with different letters are significantly different (*p* ≤ 0.05), values are means ± SD (*n* = 3)

**Table 7:** The activity of different concentrations of synthesized compounds against unicellular fungus, *Candida albicans.* Data are recorded as a zone of Inhibition (ZOI) by mm

Concentrations (µg mL^–1^)/ ZOIs (mm)				
Treatment	100	50	25	12.5
Fluconazole (Positive control)	17.3 ± 0.6^c^	11.3 ± 0.6^c^	9.7 ± 0.6^b^	0.0 ± 0.0^c^
**2a**	17.3 ± 1.2^c^	14.0 ± 1.7^b^	9.0 ± 1.0^b^	0.0 ± 0.0^c^
**2b**	24.3 ± 0.6^a^	15.3 ± 0.6^b^	11.3 ± 0.6^b^	0.0 ± 0.0^c^
**3a**	17.0 ± 1.0^c^	14.7 ± 0.6^b^	10.3 ± 0.6^b^	0.0 ± 0.0^c^
**3b**	15.7 ± 0.6^cd^	14.3 ± 0.6^b^	11.3 ± 0.6^b^	0.0 ± 0.0^c^
**4a**	0.0 ± 0.0^f^	0.0 ± 0.0^f^	0.0 ± 0.0^c^	0.0 ± 0.0^c^
**4b**	15.3 ± 1.2^cd^	11.0 ± 1.0^c^	0.0 ± 0.0^c^	0.0 ± 0.0^c^
**5a**	9.3 ± 0.6^e^	0.0 ± 0.0^f^	0.0 ± 0.0^c^	0.0 ± 0.0^c^
**5b**	20.7 ± 0.6^b^	18.3 ± 0.6^a^	13.7 ± 1.2^ab^	0.0 ± 0.0^c^

Values within the same column with different letters are significantly different (*p* ≤ 0.05), values are means ± SD (*n* = 3)

## Conclusion

In conclusion, this study successfully synthesized and characterized a series of 1,2,4-triazolo[4,3-*a*] pyrimidin-5-(1*H*)-one derivatives. The structural and electronic properties of the compounds were investigated through spectroscopic analysis and DFT calculations, revealing their planar conformations and the influence of substituents on their geometry and electronic behavior. The calculated binding energies and IR spectra confirmed the stability of the compounds, while UV-Vis’s absorption spectra demonstrated their optical properties, including a redshift due to electron-donating substituents. Density of state and HOMO/LUMO analyses provided insights into orbital interactions and electron delocalization. Moreover, the synthesized compounds exhibited promising antimicrobial potential, suggesting their potential as effective agents against microbial resistance. Overall, these findings contribute to our understanding of the derivatives’ properties and highlight their potential applications in medicinal chemistry and materials science, paving the way for further research and development.

## Electronic Supplementary Material

Below is the link to the electronic supplementary material.

Supplementary Material 1

## Data Availability

No datasets were generated or analysed during the current study.

## Declarations

### Version Number and URL Link for Software

We used Gaussian 16, Revision C.01, for all computational studies (Gaussian 16, Revision C.01, Gaussian, Inc., Wallingford CT, 2016). The software is available at https://gaussian.com. For molecular docking studies, we utilized AutoDock 4.2.6, a suite of automated docking tools that allows flexible ligand docking. AutoDock is freely available under the GNU General Public License at http://autodock.scripps.edu.

### Supplementary Information

The supplementary file accompanying this study contains calculated IR spectra, ^1^HNMR, ^1^H NMR D_2_O, ^13^C NMR and mass spectrum for all studied compounds.
