# Supplementary material for: Design, Synthesis, Antimicrobial Evaluation, Molecular Docking, and Computational Studies of New 1,2,4-Triazolo[4,3-a]pyrimidin-5(1 H)-one Derivatives
Source: J Fluoresc. 2025 Jun 30;35(11):11897–914. doi: 10.1007/s10895-025-04412-w (PMC12718226; doi:10.1007/s10895-025-04412-w)

**Supplementary Data**

**Design, Synthesis, Antimicrobial Evaluation, Molecular Docking, and Computational Studies of New 1,2,4-Triazolo[4,3-a]pyrimidin-5(1H)-one Derivatives**

**Abd-Allah S. El-Etrawy^1,2^, Noha Ryad^2^, Amr Fouda^3^, Farag F. Sherbiny^4^ , Adel. A. H. Abdel-Rahman^5^ and Mahmoud A. S. Sakr^1^**

^1^Department of Chemistry, Center of Basic Science (CBS), Misr University for Science and Technology (MUST), Al-Motamayez District, 6th of the October City 77, Egypt.

^2^Pharmaceutical Organic Chemistry Department, College of Pharmaceutical Sciences

and Drug Manufacturing, Misr University for Science and Technology (MUST), 6th of

October City, P.O. Box 77, Giza, Egypt

^3^Botany and Microbiology Department, Faculty of Science, Al-Azhar University, Nasr City, Cairo 11884, Egypt

^4^Pharmaceutical Organic Chemistry Department, Faculty of Pharmacy (Boys), Al-Azhar University, Cairo 11884, Egypt

^5^Department of Chemistry, Faculty of Science, Menoufia University, Shebin El-Koam, Egypt

**Corresponding Authors:** [mahmoud.sakr@must.edu.eg](mailto:mahmoud.sakr@must.edu.eg) (Mahmoud A.S. Sakr), and abdallah.etrawy@must.edu.eg (Abd-Allah S. El-Etrawy).

**(Fig. S1**) General procedure for synthesis of target compounds (**2-5**)

IR of Compound 2a


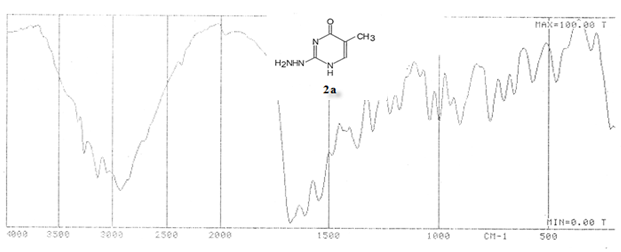


^1^H NMR of Compound 2a


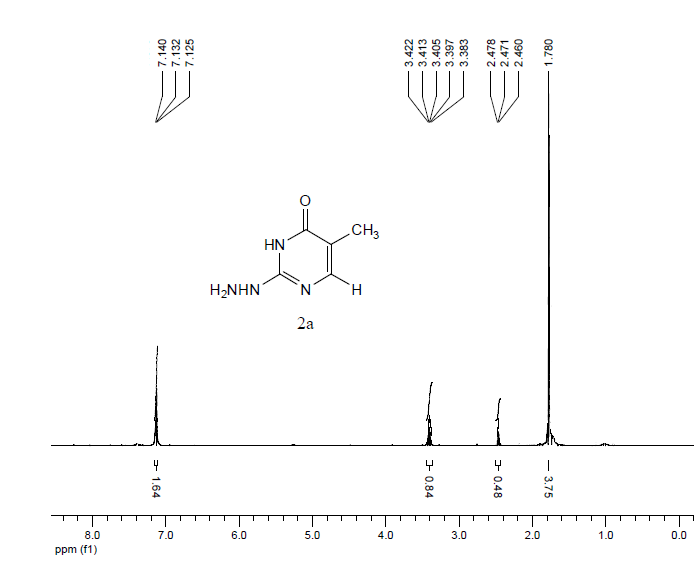


^1^H NMR D_2_O of Compound 2a


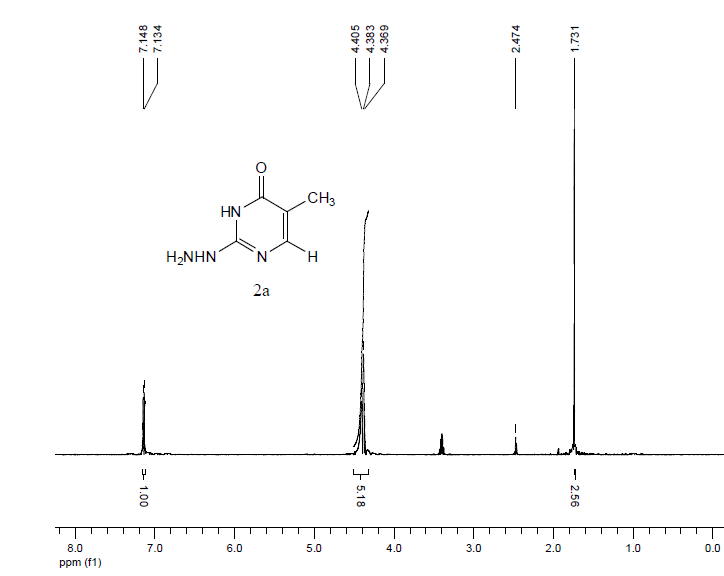


^13^C NMR of Compound 2a


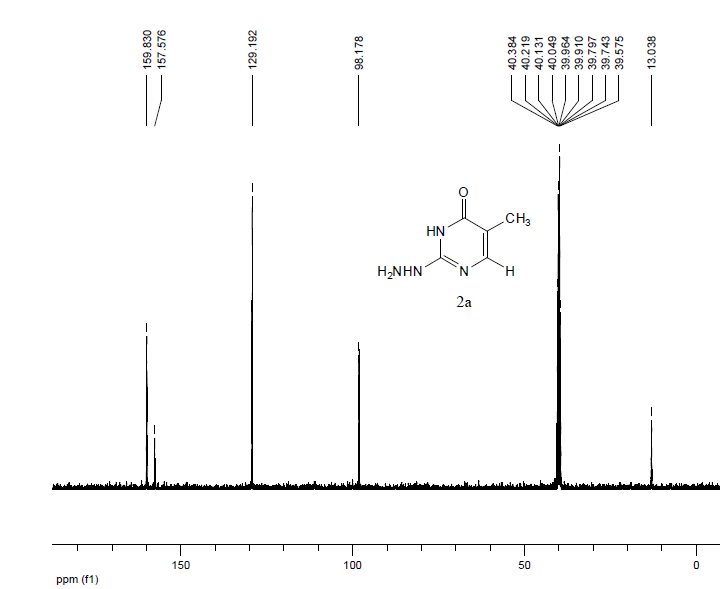


Mass spectroscopy of Compound 2a


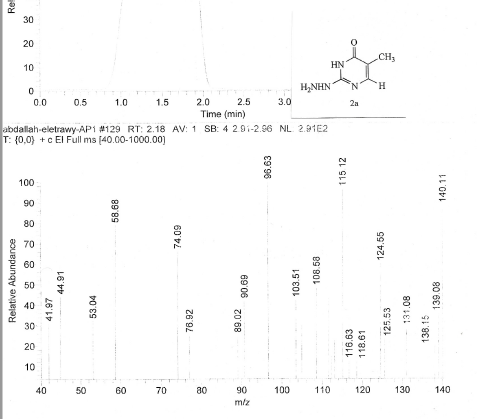


IR of Compound 2b


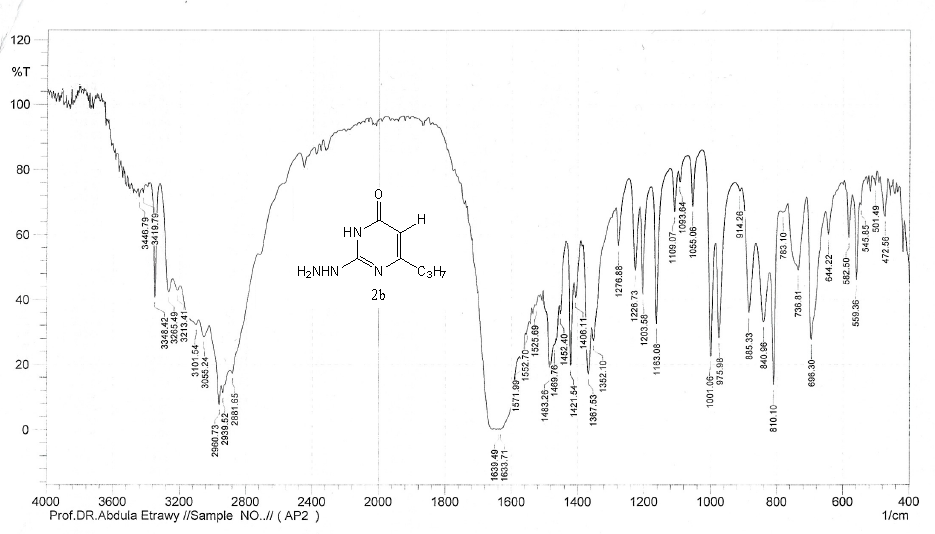


^1^H NMR of Compound 2b


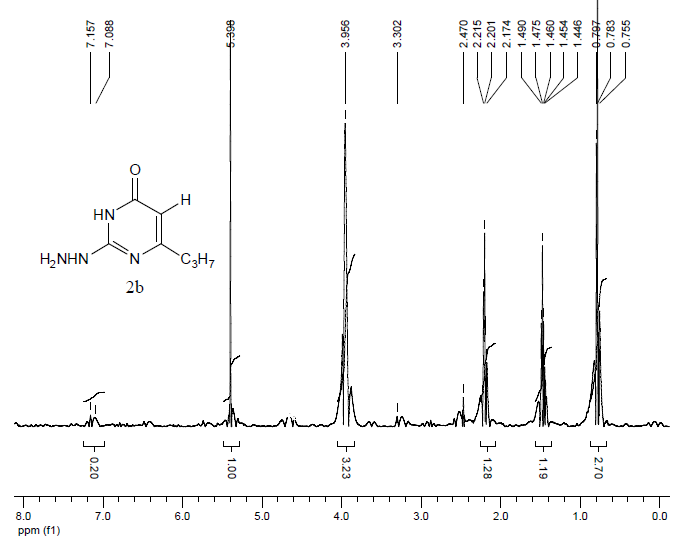


^1^H NMR D_2_O of Compound 2b


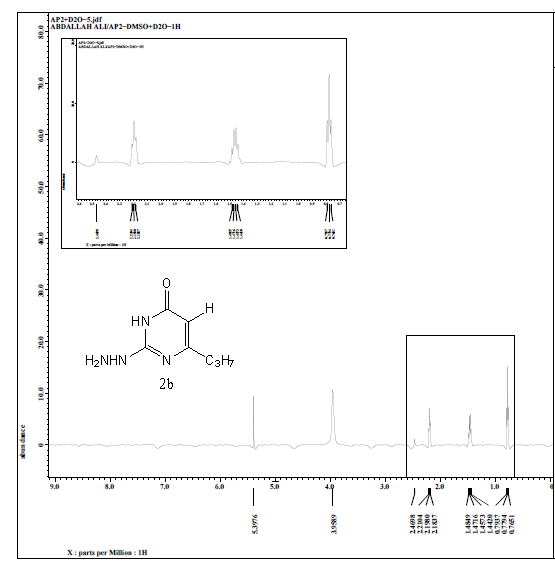


^13^C NMR of Compound 2b


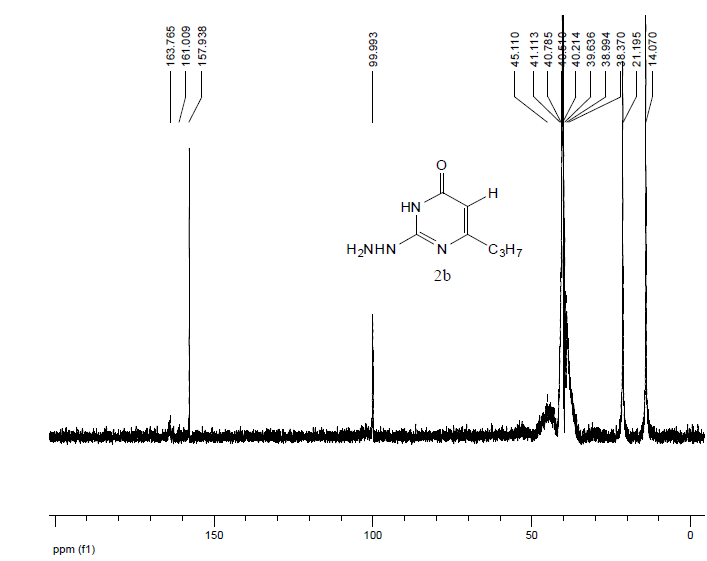


Mass spectroscopy of Compound 2b


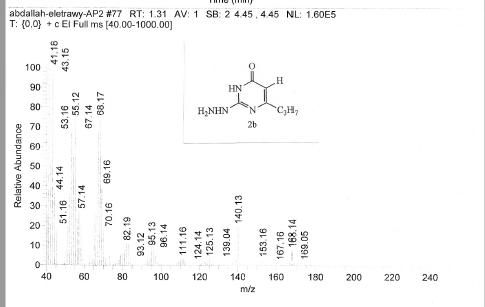


IR of Compound 3a


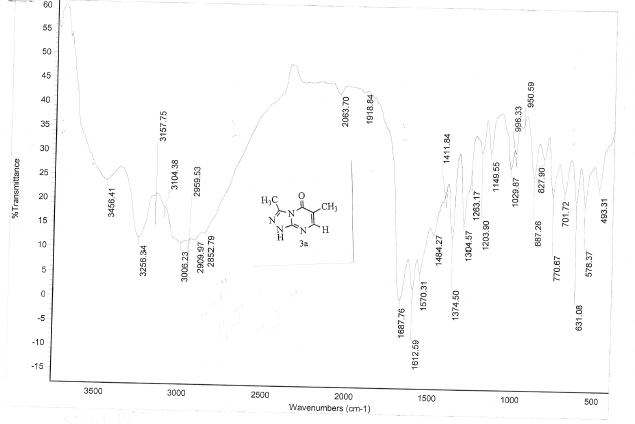


^1^H NMR of Compound 3a


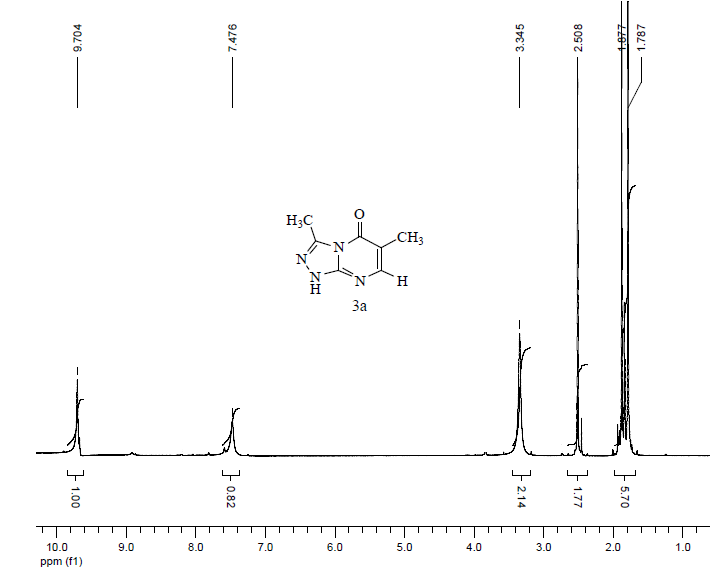


^13^C NMR of Compound 3a


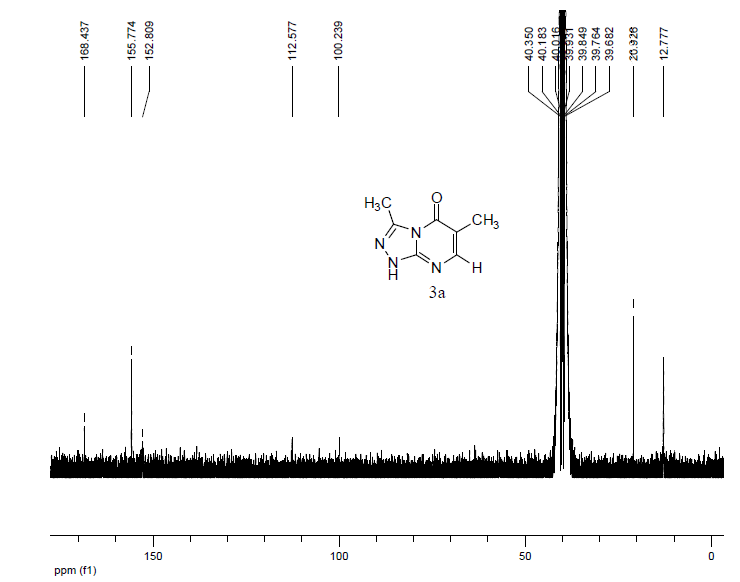


Mass spectroscopy of Compound 3a


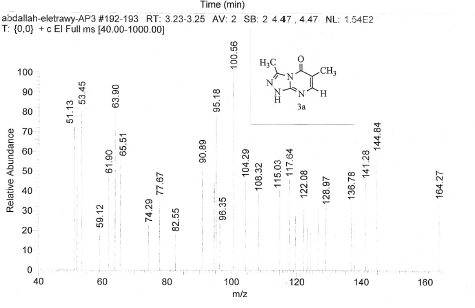


IR of Compound 3b


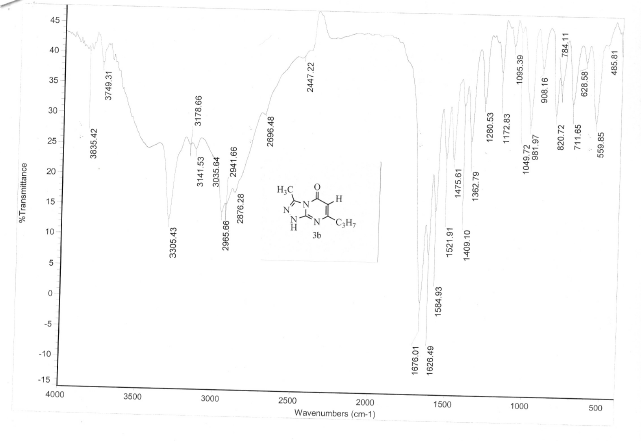


^1^H NMR of Compound 3b


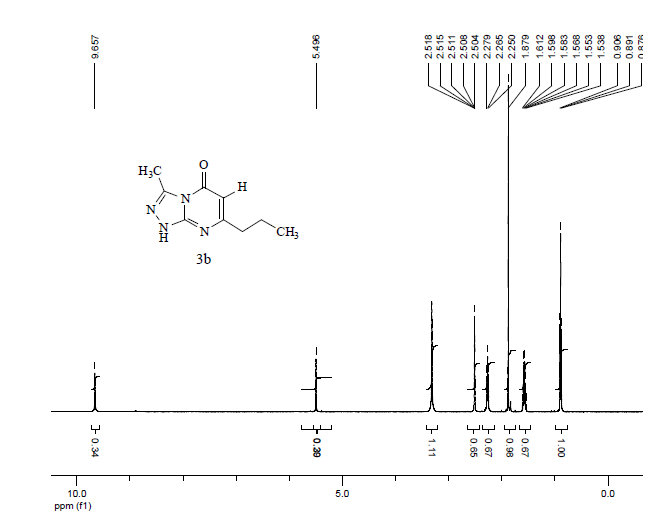


^13^C NMR of Compound 3b


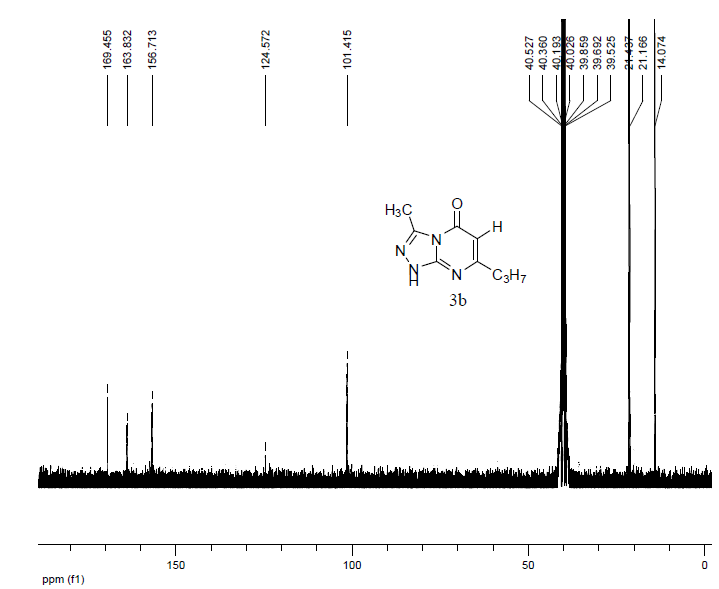


Mass spectroscopy of Compound 3b


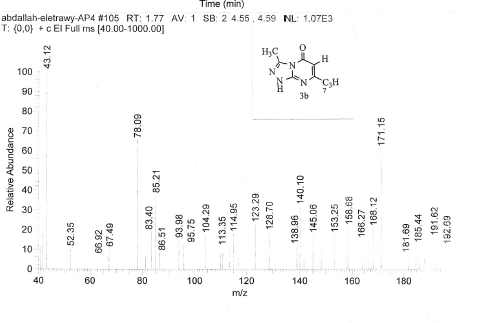


IR of Compound 4a


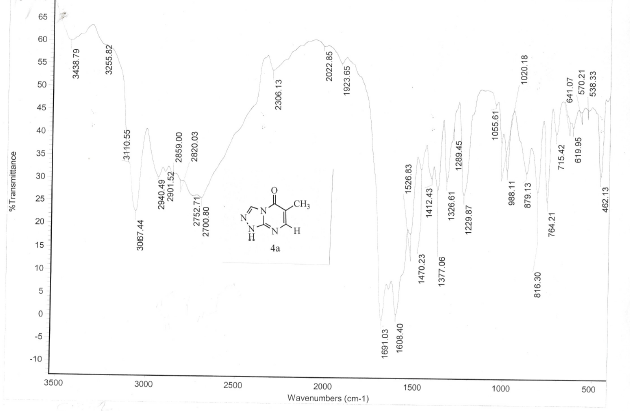


^1^H NMR of Compound 4a


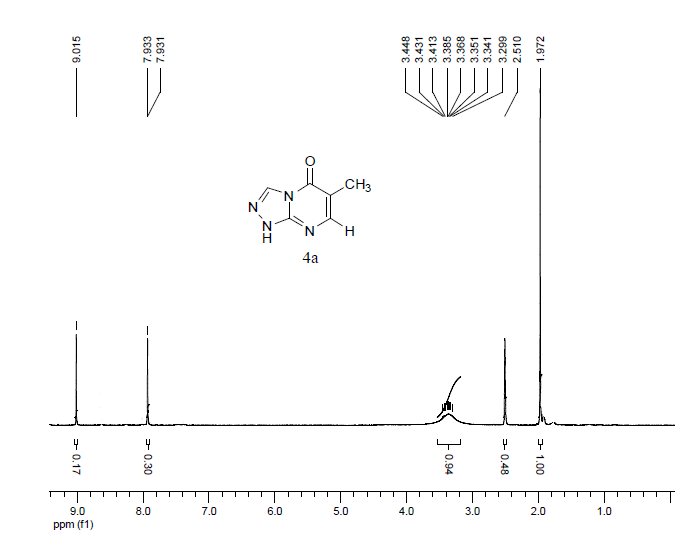


^13^C NMR of Compound4a


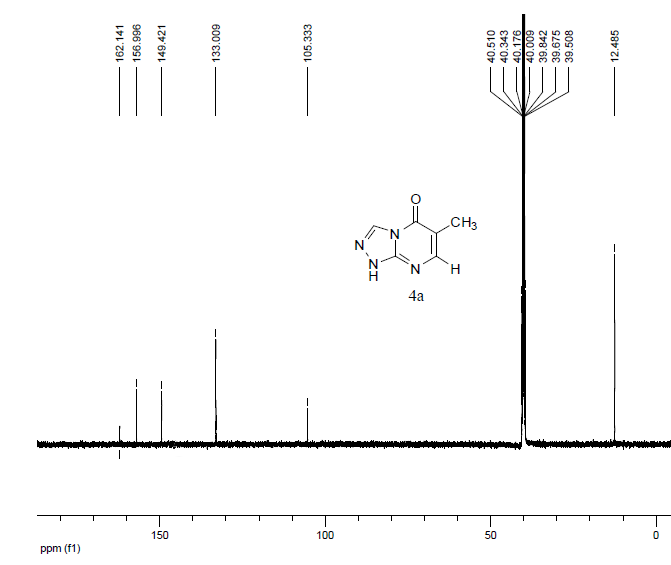


Mass spectroscopy of Compound 4a

IR of Compound 4b


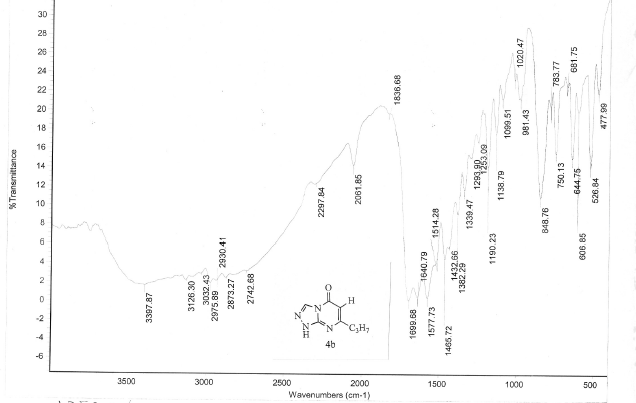


^1^H NMR of Compound 4b


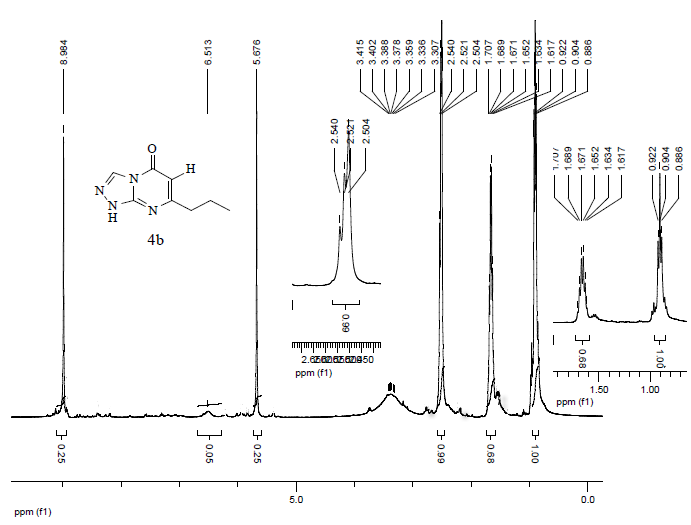


Mass spectroscopy of Compound 4b


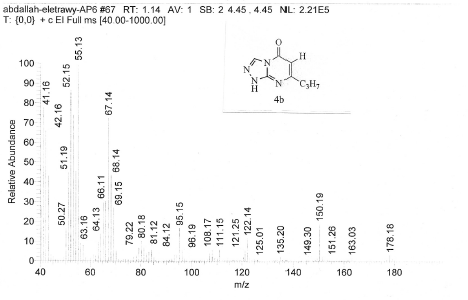


IR of Compound 5a


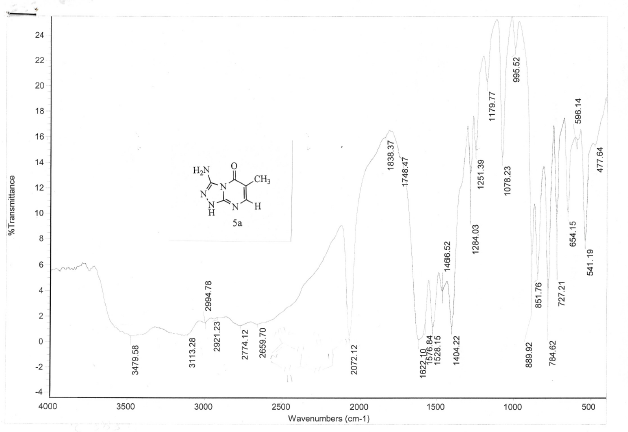


^1^H NMR of Compound 5a


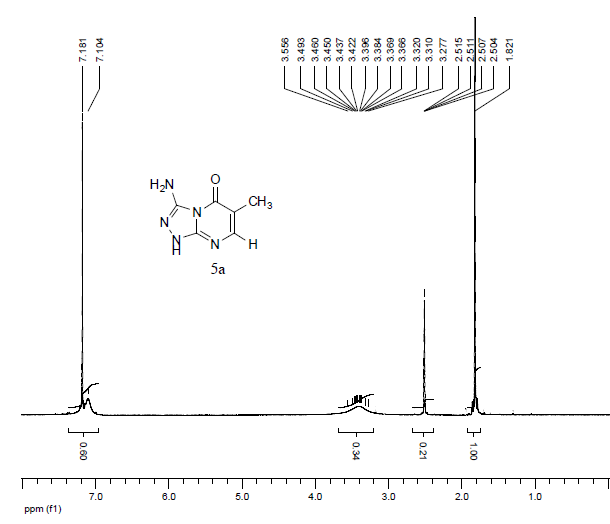


^13^C NMR of Compound 5a


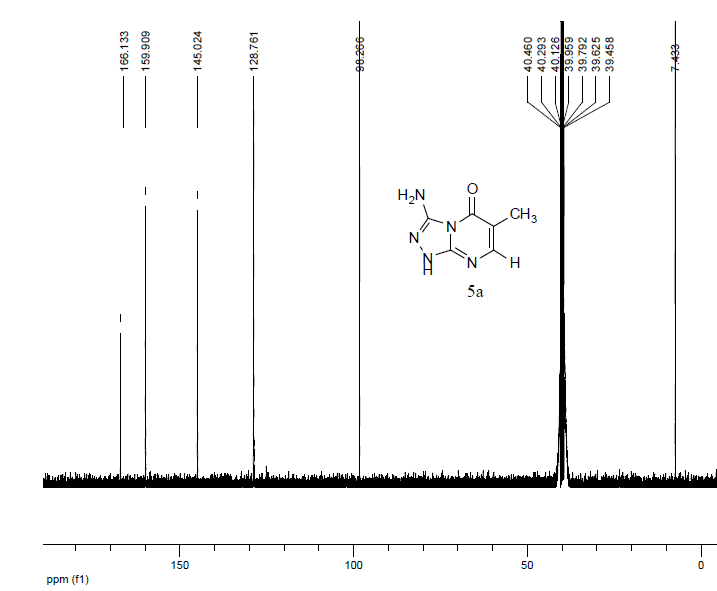


Mass spectroscopy of Compound 5a


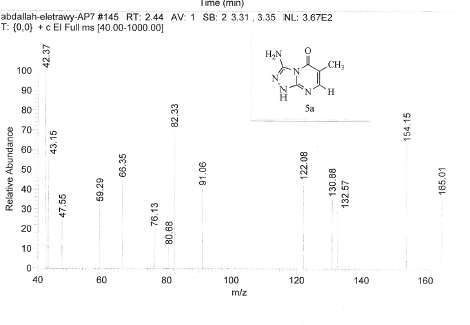


IR of Compound 5b


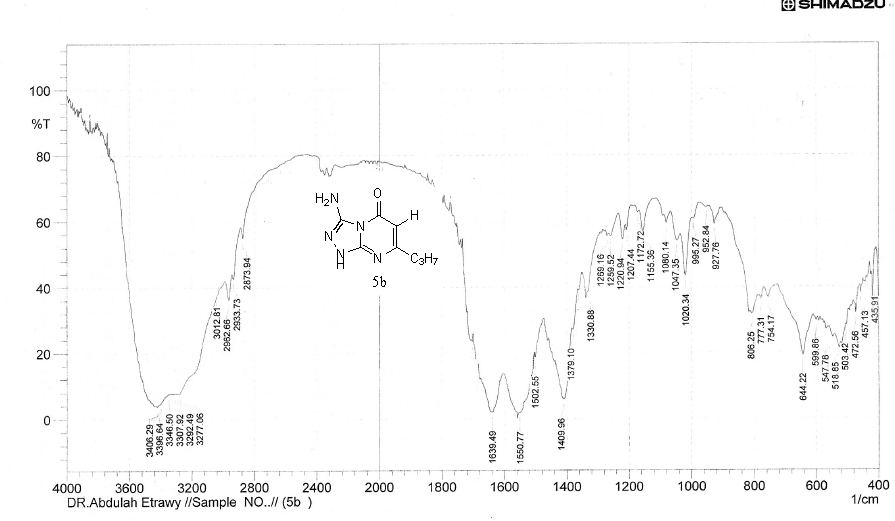


^1^H NMR of Compound 5b


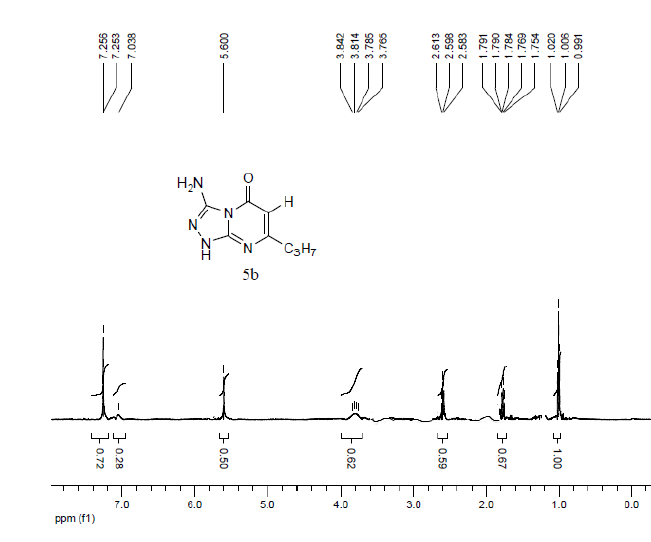


^1^H NMR D_2_O of Compound 5b


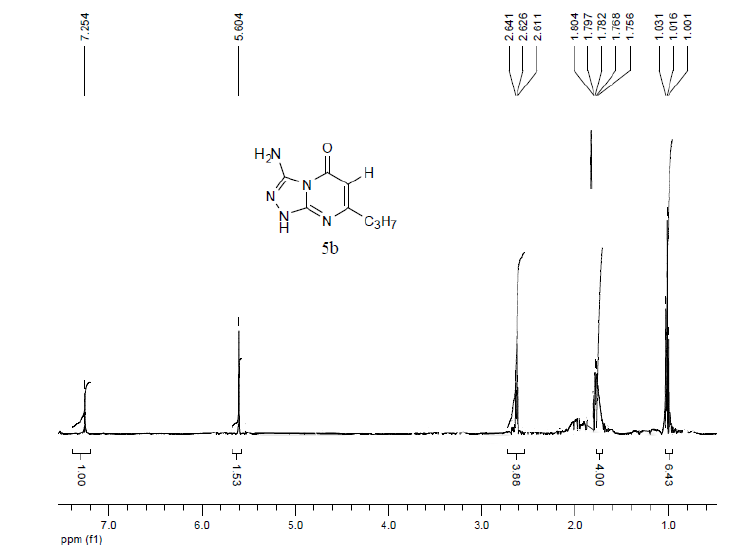


^13^C NMR of Compound 5b


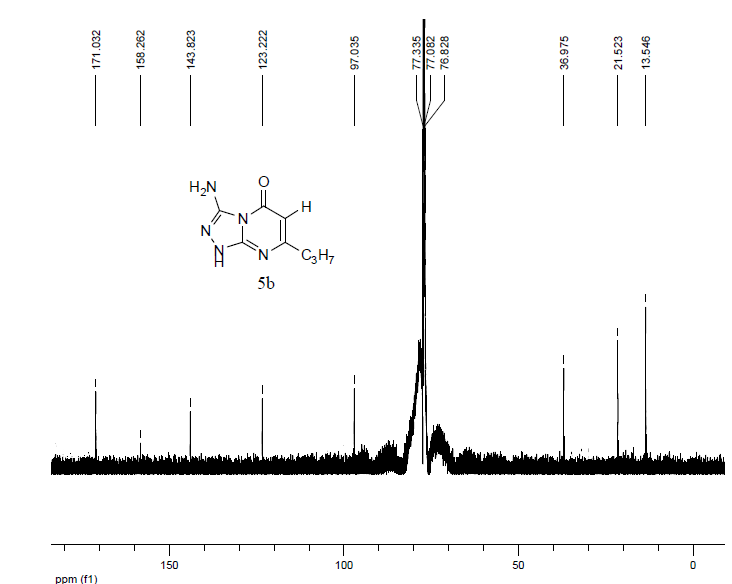


Mass spectroscopy of Compound 5b


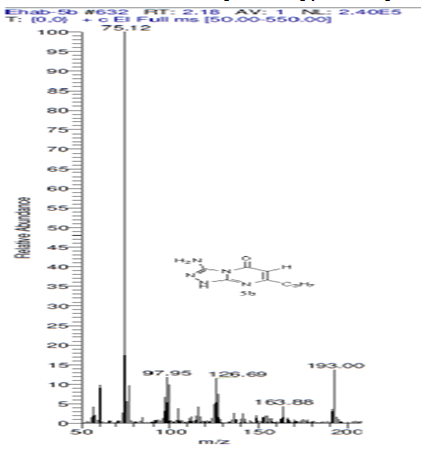

Supplement: Supplementary file 1 — Supplementary Material 1 [file 10895_2025_4412_MOESM1_ESM.docx]
